# Total chemical synthesis of a thermostable enzyme capable of polymerase chain reaction

**DOI:** 10.1038/celldisc.2017.8

**Published:** 2017-02-28

**Authors:** Weiliang Xu, Wenjun Jiang, Jiaxing Wang, Linping Yu, Ji Chen, Xianyu Liu, Lei Liu, Ting F Zhu

**Affiliations:** 1 School of Life Sciences, Tsinghua-Peking Joint Center for Life Sciences, Center for Synthetic and Systems Biology, Ministry of Education Key Laboratory of Bioinformatics, Tsinghua University, Beijing, China; 2 Department of Chemistry, Tsinghua-Peking Joint Center for Life Sciences, Ministry of Education Key Laboratory of Bioorganic Phosphorus Chemistry and Chemical Biology, Tsinghua University, Beijing, China

**Keywords:** native chemical ligation, polymerase chain reaction, protein chemical synthesis, *Sulfolobus solfataricus* P2 DNA polymerase IV, thermostable

## Abstract

Polymerase chain reaction (PCR) has been a defining tool in modern biology. Towards realizing mirror-image PCR, we have designed and chemically synthesized a mutant version of the 352-residue thermostable *Sulfolobus solfataricus* P2 DNA polymerase IV with l-amino acids and tested its PCR activity biochemically. To the best of our knowledge, this enzyme is the largest chemically synthesized protein reported to date. We show that with optimization of PCR conditions, the fully synthetic polymerase is capable of amplifying template sequences of up to 1.5 kb. The establishment of this synthetic route for chemically synthesizing DNA polymerase IV is a stepping stone towards building a d-enzyme system for mirror-image PCR, which may open up an avenue for the creation of many mirror-image molecular tools such as mirror-image systematic evolution of ligands by exponential enrichment.

## Introduction

We have recently reported the realization of mirror-image genetic replication and transcription by a chemically synthesized 174-residue d-amino-acid African swine fever virus polymerase X (ASFV pol X) system [1]. Yet, because of the poor processivity of the ASFV pol X system, the longest primer extension experiment achieved was the copying of a 44 nt Zn^2+^-dependent self-cleaving DNAzyme [1]. Additionally, because ASFV pol X is not thermostable, only a proof-of-concept replication experiment was performed by supplying fresh enzymes in each cycle [1], making it impractical for many applications that require efficient amplification of l-nucleic acid sequences, such as mirror-image systematic evolution of ligands by exponential enrichment (miSELEX) for the selection of l-nucleic acid aptamers against biological targets as potential research and therapeutic tools [2, 3].

We reasoned that a mirror-image PCR system may be realized through development of synthetic routes for other more efficient and thermostable polymerases [1]. Recent advances in synthetic organic chemistry have made it possible to chemically synthesize peptides as long as 312 residues [4]. Nonetheless, total chemical synthesis of the 832-residue *Taq* polymerase is still beyond the current technology. Thus, we set out to explore synthetic routes to chemically synthesize the 352-residue thermostable *Sulfolobus solfataricus* P2 DNA polymerase IV (Dpo4), which was shown capable of PCR-amplifying sequences longer than 1 kb in the previous study [5]. Here we have designed and chemically synthesized a mutant version of Dpo4. We showed that the fully synthetic polymerase can PCR amplify sequences from 110 bp to up to 1.5 kb, and can assemble short DNA oligonucleotides into longer gene sequences.

## Results

### Designing a mutant Dpo4 for total chemical synthesis

**Figure 1:**
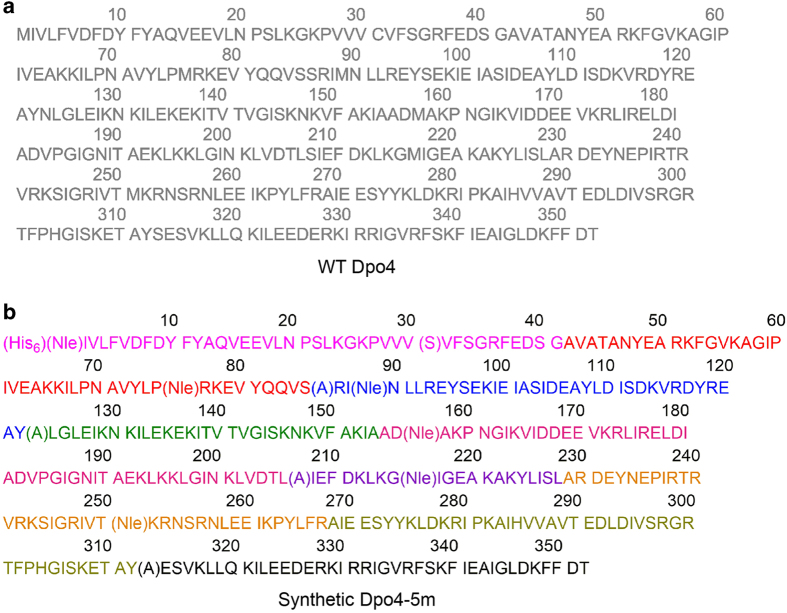
Designing a mutant Dpo4 for total chemical synthesis. (**a**) Wild-type Dpo4 amino acid sequence. (**b**) Mutant Dpo4 with His_6_ tag (Dpo4-5m) amino acid sequence with five point mutations (highlighted by parentheses) to introduce more potential ligation sites (S86A, N123A, S207A and S313A) and to avoid potential intermolecular dimerization due to disulfide bond formation in the folding process (C31S). In addition, isosteric norleucine (Nle; highlighted by parentheses) was used to replace all the methionine residues (Met1, Met76, Met89, Met157, Met216 and Met251) to avoid oxidation in the SPPS and protein ligation processes. Colors of the nine peptide segments correspond to the peptide segment colors used in Figure 2 (Dpo4-1 to Dpo4-9).

**Figure 3:**
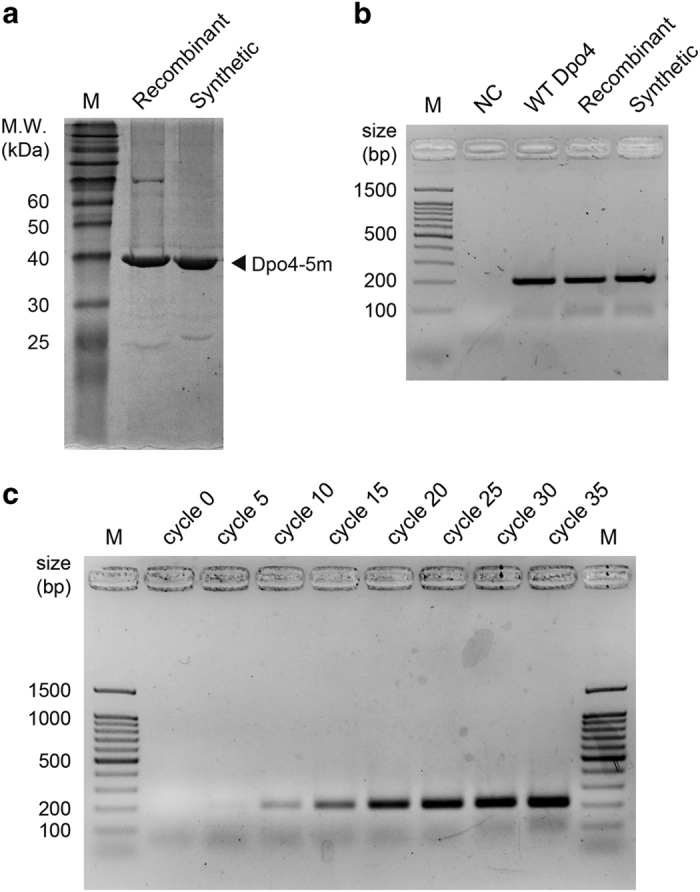
Biochemical characterization of Dpo4-5m. (**a**) Chemically synthesized and purified 40.8 kDa Dpo4-5m, as well as recombinant Dpo4-5m (with His_6_ tag) purified from the *E. coli* strain BL21(DE3) were analyzed by 12% sodium dodecyl sulfate-polyacrylamide gel electrophoresis (SDS-PAGE), stained by Coomassie Brilliant Blue. A small fraction of unligated peptide segments can be observed in the synthetic Dpo4-5m. M, protein marker. (**b**) PCR amplification of a 200 bp sequence by recombinant wild-type Dpo4 ('WT Dpo4'), recombinant Dpo4-5m ('Recombinant') and synthetic Dpo4-5m ('Synthetic'), performed in 50 mM HEPES (pH 7.5), 5 mM MgCl_2_, 50 mM NaCl, 0.1 mM EDTA, 5 mM dithiothreitol, 10% glycerol, 3% dimethyl sulfoxide, 0.1 mg ml^−1^ bovine serum albumin, 200 μM (each) ultrapure dNTPs, 0.5 μM (each) primers, 2 nM linearized dsDNA template and ~300 nM polymerase for 35 cycles. The products were analyzed by 2% agarose gel electrophoresis and stained by GoldView. NC, negative control with template and primers but without enzyme. (**c**) PCR amplification of a 200 bp sequence by synthetic Dpo4-5m, sampled from multiple cycles. The products were analyzed by 2% agarose gel electrophoresis and stained by GoldView, with cycle numbers from which they were sampled indicated above the lanes. M, DNA marker.

Because it becomes increasingly difficult to synthetize peptides longer than 50 residues directly by solid-phase peptide synthesis (SPPS), we applied the native chemical ligation (NCL) approach to ligate short peptide segments into longer ones through native peptide bonds [6, 7]. NCL requires an N-terminal Cys residue at the ligation site, and yet only one cysteine (Cys31) is present in the wild-type (WT) Dpo4 amino acid sequence (Figure 1a). To use the four alanine residues in Dpo4 (A42, A155, A229 and A268) as ligation sites, we took advantage of a metal-free radical-based desulfurization approach that can convert unprotected Cys to Ala after NCL [8]. However, even with these four ligation sites, most of the peptide segments were still more than 50 amino acids. Thus, we designed a mutant version of the Dpo4 enzyme consisting of four point mutations (S86A, N123A, S207A and S313A) to introduce more ligation sites to the enzyme’s amino acid sequence (Figure 1b). Moreover, we also introduced a C31S mutation to avoid potential intermolecular dimerization due to disulfide bond formation in the folding process. We experimentally tested the activity of the mutant Dpo4 enzyme using recombinant polymerase purified from *Escherichia coli*, which suggested that the presence of the five point mutations (C31S, S86A, N123A, S207A and S313A) did not adversely affect the PCR activity of the enzyme (Figure 3b).

**Figure 2:**
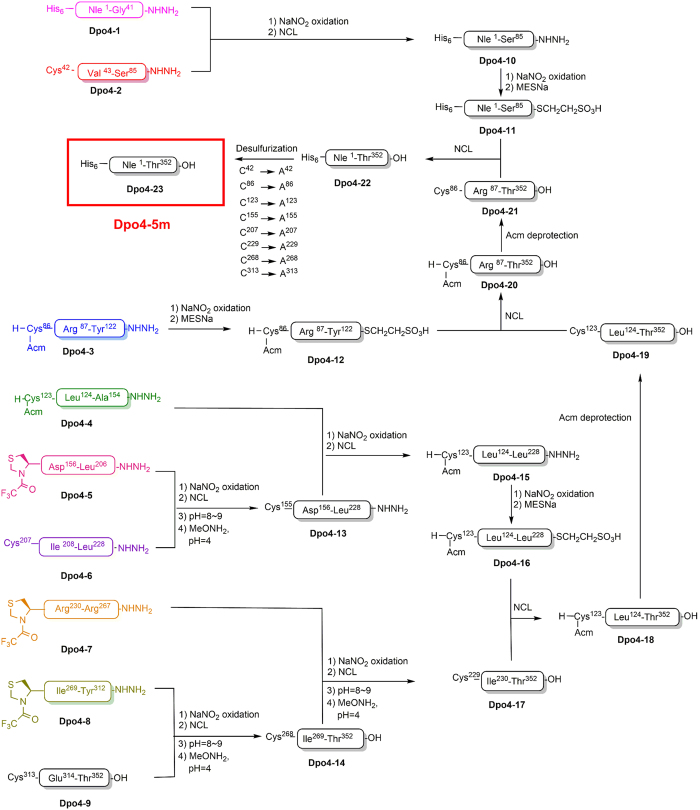
Synthetic route for synthesizing Dpo4-5m. Total chemical synthesis of Dpo4-5m by assembling nine peptide segments in the C- to N-terminus direction using hydrazides as thioester surrogates.

With these five point mutations, the total chemical synthesis of Dpo4-5m can be achieved by assembling nine peptide segments (Dpo4-1 to Dpo4-9) in the C- to N-terminus direction using hydrazides as thioester surrogates (Figure 2) [7, 9, 10]. In addition, we replaced all the methionine residues (Met1, Met76, Met89, Met157, Met216 and Met251) with norleucine to avoid potential oxidation in the SPPS and protein ligation processes. Norleucine is isosteric with methionine and is expected to have little effect on the protein structure and function [11]. We also added a polyhistidine tag (His_6_) at the N terminus of the synthetic polymerase to facilitate further protein purification after folding of the enzyme (see Materials and Methods section), making the total length of the synthetic peptide (Dpo4-5m) to 358 amino acids (352 amino-acid polymerase plus 6 amino-acid His_6_ tag; see Figure 1b).

### Chemical synthesis of Dpo4-5m

All the peptide segments ranging in lengths from 22 to 52 amino acids were synthesized by Fmoc-based SPPS and purified by reversed-phase high-performance liquid chromatography (RP-HPLC). During the SPPS, we discovered that the crude peptide segments Dpo4-1 and Dpo4-3 had poor solubility in aqueous acetonitrile solutions because of their high hydrophobicity. It has been known that incorporation of isoacyl dipeptides into peptide segments can help improve the solubility of peptides in aqueous solutions [12, 13]. Thus, we inserted isoacyl dipeptides at positions Val30-Ser31 (in segment Dpo4-1) and Ala102-Ser103 (in segment Dpo4-3), which worked as traceless modifications due to rapid O-to-N acyl shift under NCL conditions at pH ~7 [12, 13]. Our results suggested that the incorporation of isoacyl dipeptides improved not only the solubility but also the purity of the crude peptide segments (data not shown). In addition, we used acetamidomethyl, a Cys-protecting group that prevents cyclization of peptides during ligation and Cys desulfurization [14], to protect the N-terminal Cys in Dpo4-3 and Dpo4-4. Meanwhile, we incorporated trifluoroacetyl thiazolidine-4-caboxylic acid, which is stable during hydrazide oxidation and can quickly convert back to Thz in aqueous base conditions [15], into Dpo4-5, Dpo4-7 and Dpo4-8 as N-terminal Cys-protecting group. Therefore, the N-terminal Cys-unprotected segments (Dpo4-13, Dpo4-14 and Dpo4-17) were conveniently obtained by one-pot ligation and trifluoroacetyl thiazolidine-4-caboxylic acid deprotection (see Supplementary Information).

After the assembly of nine peptide segments in the C- to N-terminus direction using hydrazides as thioester surrogates and HPLC purification [7, 9, 10], we obtained the 358 amino-acid synthetic peptide with a yield of ~16 mg (Supplementary Figure S1-14), which was subject to the folding, heating to precipitate thermolabile peptides, Ni-NTA column purification and concentration steps as described below. We analyzed the full-length peptide by analytical HPLC chromatogram and electrospray ionization mass spectrometry to validate its molecular weight (observed MW 40 797.5 Da, calculated MW 40 799.9 Da) (Supplementary Figure S14B and C). The lyophilized peptide was dissolved in a denaturation buffer containing 6 m Gn·HCl, and folded by successive dialysis against a series of renaturation buffers containing 4, 2, 1, 0.5, 0.25 and 0 m Gn·HCl, respectively. We then heated the folded polymerase to 78 °C to precipitate the thermolabile peptides, which were subsequently removed by ultracentrifugation. The thermostable synthetic Dpo4-5m in the supernatant was further purified by a Ni-NTA column and concentrated by centrifugal filter, until ~2 mg folded synthetic Dpo4-5m was obtained (observed MW 40 797.3 Da, calculated MW 40 799.9 Da), the purity of which was considerably improved compared with the unfolded product (Supplementary Figure S14D). We also analyzed the synthetic Dpo4-5m by sodium dodecyl sulfate-polyacrylamide gel electrophoresis (Figure 3a), and verified the amino acid sequence of the synthetic protein by liquid chromatography-tandem mass spectrometry, with a collective 100% coverage from two independent experiments with the peptide treated by trypsin and pepsin, respectively (Supplementary Table S2).

### PCR by synthetic Dpo4-5m

Having folded and purified the chemically synthesized Dpo4-5m, we tested the PCR activity of the enzyme with a 200 bp template (Figure 3b and c). The amplification efficiency of the synthetic polymerase, which measured ~1.5 (see Materials and Methods section; amplification efficiency estimated based on the agarose gel electrophoresis results shown in Supplementary Figure S15). This efficiency was comparable to those of the recombinant WT and mutant enzymes, which also measured ~1.5 based on the same approach. The PCR products were cloned into T-vectors for Sanger sequencing. The alignment results suggested the presence of a total of 7 single-base deletions and 19 single-base mutations among the 22 clones sequenced (Supplementary Figure S16), which corresponds to a ~0.9% accumulated mutation rate after 35 PCR cycles, consistent with the replication error rate of recombinant WT Dpo4 reported in previous studies [5, 16].

**Figure 4:**
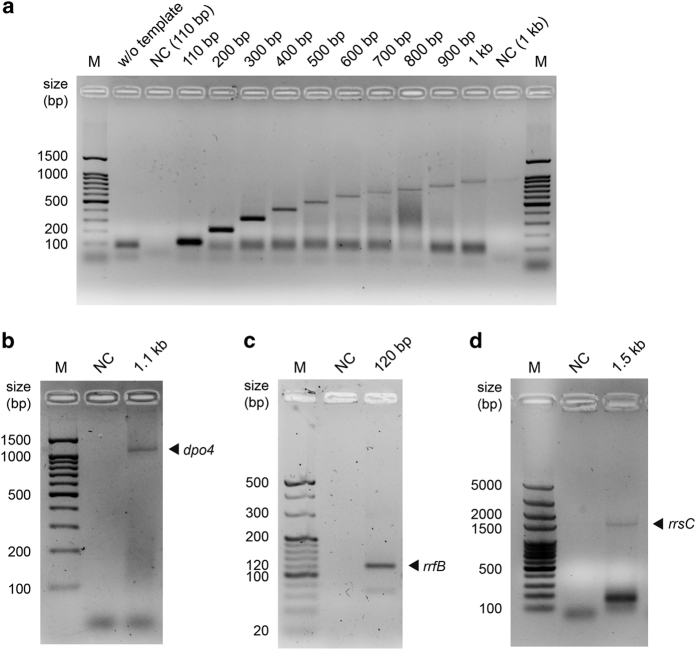
PCR amplification of various sequences by synthetic Dpo4-5m. (**a**) PCR amplification of sequences with lengths ranging from 110 bp to 1 kb by synthetic Dpo4-5m for 35 cycles. The expected amplicon lengths are indicated above the lanes. Primer dimer bands can be observed below the main product bands. The extension time was set to 2 min per cycle for 110–300 bp sequences, 5 min for 400–600 bp sequences and 10 min for 700–1000 bp sequences. W/o template: negative control with primers and enzyme but without template; NC (110 bp)/NC (1 kb): negative control with 110 bp or 1 kb templates and primers but without enzyme. (**b**) PCR amplification of the 1.1 kb *dpo4* gene by synthetic Dpo4-5 m for 35 cycles, with the extension time set to 10 min per cycle. (**c**) PCR amplification of the 120 bp 5S *E. coli* rRNA gene *rrfB* by synthetic Dpo4-5m for 35 cycles, with the extension time set to 2 min per cycle. (**d**) PCR amplification of the 1.5 kb 16S *E. coli* rRNA gene *rrsC* by synthetic Dpo4-5m for 35 cycles, with the extension time set to 15 min per cycle. A primer dimer band can be observed below the main product band. All the PCR products were analyzed by 2% agarose gel electrophoresis and stained by GoldView. The expected amplicon lengths are indicated above the lanes. NC, negative control with template and primers but without enzyme. M, DNA marker.

Next, we tested the PCR activity of synthetic Dpo4-5m with templates of varying lengths. We show that the synthetic polymerase is capable of amplifying sequences ranging in lengths from 110 bp to 1 kb in 35 cycles (Figure 4a), although the amplification appeared to be less efficient with longer templates. The PCR conditions were similar in the experiments, except that the extension time was set to 2 min per cycle for 110–300 bp sequences, 5 min for 400–600 bp sequences and 10 min for 700–1000 bp sequences (see Materials and Methods section).

Encouraged by the successful PCR amplification of sequences as long as 1 kb, we carried out PCR amplification of the 1.1 kb *dpo4* gene coding for the polymerase itself (Figure 4b). Additionally, we successfully amplified the 120 bp 5S *E. coli* ribosomal RNA (rRNA) gene *rrfB* (Figure 4c), as well as the 1.5 kb 16S *E. coli* rRNA gene *rrsC* (Figure 4d). The ability of synthetic Dpo4-5m to amplify rRNA genes is significant because they can be used for the transcription of rRNAs in the assembly of a functional ribosome [17]. This 1.5 kb amplicon is even longer than the 1.3 kb sequence previously amplified by the recombinant WT Dpo4 [5], although longer templates might have not been tested in prior studies.

### Assembly PCR by synthetic Dpo4-5m

**Figure 5:**
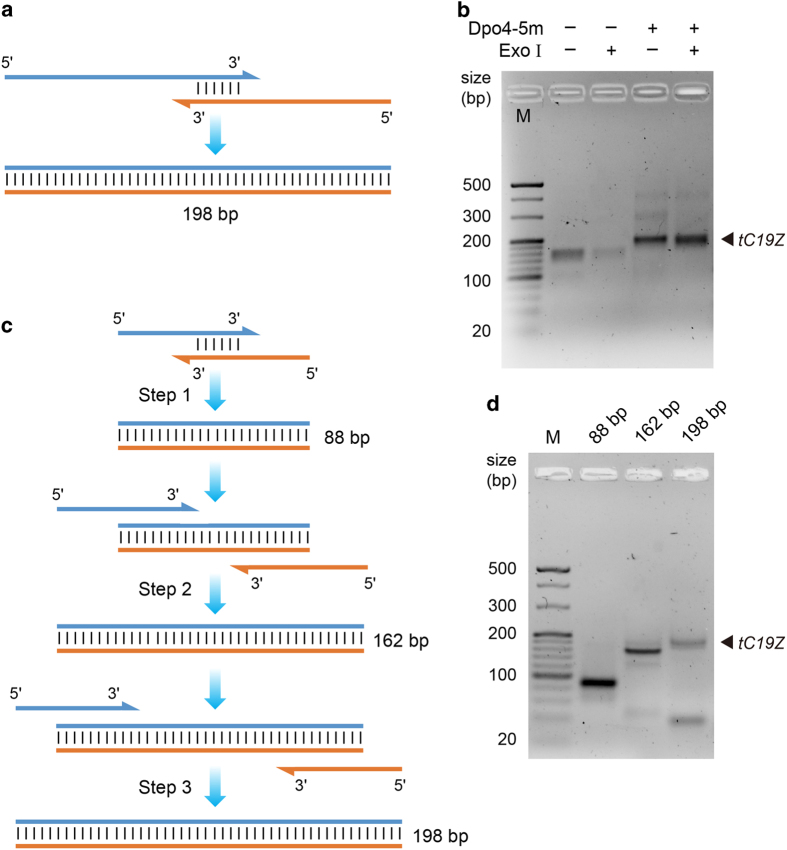
Assembly PCR by synthetic Dpo4-5m. (**a**, **b**) Assembly PCR using two long primers (*tC19Z*-F115 and *tC19Z*-R113) with a 30 bp sequence overlap for 20 cycles to obtain the 198 bp *tC19Z* gene, analyzed by 3% sieving agarose gel electrophoresis and stained by GoldView. Exo I: treated by exonuclease I (which digests single-stranded DNA (ssDNA) but not dsDNA). (**c**, **d**) Three-step assembly PCR using six short primers ranging in lengths from 47 to 59 nt to obtain the 198 bp *tC19Z* gene, analyzed by 3% sieving agarose gel electrophoresis and stained by GoldView. The PCR cycle numbers for steps 1, 2 and 3 were 5, 10 and 20, respectively. The expected amplicon lengths of each step (88, 162 and 198 bp, respectively) are indicated above the lanes. Primer dimer bands can be observed below the main product bands. M, DNA marker.

Assembly PCR is a convenient way to generate sequences longer than chemically synthesized DNA oligonucleotides (typically shorter than 150 nt) [18]. This is of particular importance for gene assembly in a future mirror-image PCR system, considering the lack of available l-DNA template sequences in nature. Here we tested the ability of synthetic Dpo4-5m to assemble the 198 bp *tC19Z* gene coding for an *in vitro* evolved RNA polymerase ribozyme [19], using two long primers (*tC19Z*-F115 and *tC19Z*-R113) with a 30 bp sequence overlap (Figure 5a). After 20 cycles of amplification, both the amplified and negative control groups were treated by exonuclease I (which digests single-stranded DNA but not double-stranded DNA (dsDNA)). Agarose gel electrophoresis results suggested that the full-length 198 bp dsDNA product was successfully assembled (Figure 5b), and its sequence was verified by Sanger sequencing.

We also experimented with the assembly of the 198 bp *tC19Z* gene using six short primers ranging in lengths from 47 to 59 nt through three steps of assembly PCR (Figure 5c), a similar strategy of which could be used to obtain long mirror-image gene sequences from multiple short l-DNA oligonucleotides in a future mirror-image PCR system. Step 1 was performed by assembly PCR with primers *tC19Z*-F1 and *tC19Z*-R1, the product of which was further amplified by *tC19Z*-F2 and *tC19Z*-R2 in step 2, and finally the product was amplified by *tC19Z*-F3 and *tC19Z*-R3 in Step 3. The PCR cycle numbers for steps 1, 2 and 3 were 5, 10 and 20, respectively (see Materials and Methods section). The assembly PCR products from each step (88, 162 and 198 bp, respectively) were analyzed by agarose gel electrophoresis (Figure 5d), and the full-length product sequence was verified by Sanger sequencing. We also attempted to perform assembly PCR with all six primers in a one-step reaction, but the assembly was complicated by the existence of multiple by-product bands.

## Discussion

Our previously reported mirror-image genetic replication system based on ASFV pol X lacked the processivity and thermal stability to PCR amplify l-DNA sequences [1]. Here we have designed and verified a synthetic route for the total chemical synthesis of a mutant version Dpo4 capable of PCR, paving the way for the realization of a mirror-image PCR system. An efficient mirror-image PCR system may lead to the development of many new mirror-image molecular tools. For example, a chirally inverted PCR system may enable miSELEX for the selection of l-nucleic acid aptamers against important biological targets [2, 3].

One of the potential obstacles in the development of mirror-image PCR tools is the lack of long l-DNA template sequences. The ability of synthetic Dpo4-5m to perform assembly PCR with short chemically synthesized oligonucleotides as we have demonstrated here may provide a potential solution. However, as PCR by synthetic Dpo4-5m appeared to be less efficient with longer sequences, to obtain mirror-image genes longer than 1 kb (e.g., the 16S and 23S rRNA genes) may still be difficult. The development of efficient mirror-image DNA or RNA ligation systems, through either total chemical synthesis of a d-amino-acid ligase enzyme or utility of a cross-chiral ligase ribozyme [20], may help address this problem. In addition, DNA polymerases often possess lower PCR performance with GC-rich templates, and this may also be true with Dpo4 based on previous kinetic studies on the enzyme [21]. In the current study, we have attempted to optimize the PCR conditions by adding dimethyl sulfoxide, adding proline, varying Mg^2+^ concentrations and varying temperature conditions. We discovered that the addition of glycerol helped to improve the PCR efficiency, which was used in all of our PCR experiments. Future optimization of PCR conditions can be carried out particularly for the amplification of longer templates.

Although the synthetic route we have developed here has led to the chemical synthesis of Dpo4-5m, it is still not a practical way for the industrial scale production of d-enzymes. Future efforts to reduce the cost of synthesis could be achieved by further optimization of the synthetic route with the use of other alternative ligation strategies [22–24], or by designing truncated versions of Dpo4 for total chemical synthesis.

Another strategy towards realizing efficient mirror-image PCR is to look beyond Dpo4 for other polymerase systems. Besides its low amplification efficiency with long sequences, Dpo4 is also known to be error-prone, with its replication error rate in the range of 4×10^−4^–8×10^−3^ [5, 16], which may not be sufficient for obtaining the 16S and 23S rRNA genes by assembly PCR. One may look into the thermophile polymerase catalog for other small PCR-competent polymerases as models for building an efficient mirror-image PCR system. This goal may also be achieved by the directed evolution of small polymerases for more efficient, thermostable polymerase mutants [25, 26].

## Materials and Methods

### Fmoc-based solid-phase peptide synthesis

All the peptide segments were synthesized manually. Amino acid coupling was carried out using either four equivalent (eq.) Fmoc amino acid, 4 eq. ethyl cyanoglyoxylate-2-oxime and 4 eq. *N*,*N′*-diisopropylcarbodiimide in dimethylformamide or 4 eq. Fmoc amino acid, 3.8 eq. *O*-(6-chloro-benzotriazol-1-yl)-*N*,*N*,*N′*,*N′*-tetramethyluronium hexafluorophosphate and 8 eq. *N*,*N*-diisopropylethylamine in dimethylformamide. Air-bath heating was used to accelerate the reaction when needed [27]. Peptide Dpo4-9 was synthesized on Fmoc-Thr(tBu)-Wang resin (GL Biochem, Shanghai, China). Other peptides were synthesized on Fmoc-hydrazine 2-chlorotrityl chloride resin to prepare peptide hydrazides [28]. Val30-Ser31 (in segment Dpo4-1) and Ala102-Ser103 (in segment Dpo4-3) were coupled as isoacyl dipeptides. Trifluoroacetyl thiazolidine-4-caboxylic acid-OH was coupled using ethyl cyanoglyoxylate-2-oxime/*N*,*N′*-diisopropylcarbodiimide activation at room temperature. After the completion of peptide chain assembly, peptides were cleaved from resin using H_2_O/thioanisole/triisopropylsilane/1,2-ethanedithiol/trifluoroacetic acid (0.5/0.5/0.5/0.25/8.25). After N_2_ bubbling, Et_2_O precipitation and centrifugation, the crude peptides were obtained.

### Native chemical ligation

The C-terminal peptide hydrazide segment was dissolved in acidified ligation buffer (aqueous solution of 6 m Gn·HCl and 0.1 m NaH_2_PO_4_, pH 3.0). The mixture was cooled in an ice-salt bath (−15 °C), and 10–20 eq. NaNO_2_ in acidified ligation buffer (pH 3.0) was added. The activation reaction system was kept in ice-salt bath under stirring for 30 min, after which 40 eq. 4-mercaptophenylacetic acid in ligation buffer and 1 eq. N-terminal Cys peptide were added, and the pH of the solution was adjusted to 6.5 at room temperature. After overnight reaction, 100 mM Tris(2-carboxyethyl)phosphine hydrochloride in pH 7.0 ligation buffer was added to dilute the system twice and the reaction system was kept at room temperature for 1 h under stirring. Finally, the ligation product was analyzed by HPLC and electrospray ionization mass spectrometry, and purified by semipreparative HPLC.

### Protein folding and purification

Lyophilized Dpo4-5m was dissolved in denaturation buffer containing 6 m Gn·HCl, and dialyzed against a series of renaturation buffers which contained 4, 2, 1, 0.5, 0.25 and 0 m Gn·HCl, respectively. Each step of the dialysis was carried out at 4 °C for 10 h with gentle stirring. The denaturation and renaturation buffers also contained 50 mM Tris-acetate (pH 7.5), 50 mM NaAc, 1 mM dithiothreitol, 0.5 mM EDTA and 16% glycerol. After renaturation, the enzyme was dialyzed against a buffer containing 10 mM potassium phosphate buffer (pH 7.0), 50 mM NaCl, 10 mM MgAc_2_, 10% glycerol and 0.1% 2-mercaptoethanol. The folded polymerase was heated to 78 °C for 12 min to precipitate the thermolabile peptides, which were subsequently removed by ultracentrifugation at 19 000 r.p.m. for 40 min at 4 °C. The supernatant was incubated in Ni-NTA Superflow resin (Qiagen, Venlo, Netherlands) overnight at 4 °C, and purified according to previously described methods but without the use of Mono S column [21]. The concentration of the purified Dpo4-5m was measured spectrophotometrically at 280 nm using an extinction coefficient of 24 058 m^−1^cm^−1^ and calculated MW of 40.8 kDa. Approximately 100 μg of the purified synthetic polymerase was analyzed by 12% sodium dodecyl sulfate-polyacrylamide gel electrophoresis along with the purified recombinant protein.

### PCR by synthetic Dpo4-5m

Most of the PCR reactions were performed in 20 μl reaction systems containing 50 mM HEPES (pH 7.5), 5 mM MgCl_2_, 50 mM NaCl, 0.1 mM EDTA, 5 mM dithiothreitol, 10% glycerol, 3% dimethyl sulfoxide, 0.1 mg ml^−1^ BSA, 200 μM (each) ultrapure dNTPs, 0.5 μM (each) primers, 2 nM linearized dsDNA template and ~300 nM Dpo4-5m polymerase. The PCR program settings were 86 °C for 3 min (initial denaturation); 86 °C for 30 s, 58–65 °C (Tm-dependent) for 1 min and 65 °C for 2 to 15 min (depending on the amplicon length), for 35 cycles; 65 °C for 5 min (final extension). To test the PCR activity of synthetic Dpo4-5m on templates of varying lengths, the same primer pair was used (M13-long-F and M13-long-R; see Supplementary Table S1), and the extension time was set to 2 min per cycle for 110–300 bp sequences, 5 min for 400–600 bp sequences and 10 min for 700–1000 bp sequences. The PCR products were analyzed by 2% agarose gel electrophoresis and stained by GoldView (Solarbio). To quantify the amplification efficiency and fidelity of synthetic Dpo4-5m, a linear 200 bp DNA sequence prepared by Q5 DNA polymerase (New England Biolabs, Ipswitch, MA, USA) followed by gel purification was used as template. The bands of the first 10 cycles were analyzed by the Image Lab software (Bio-Rad, Hercules, CA, USA). The PCR product after cycle 35 was purified by the DNA Clean and Concentrator Kit (Zymo Research, Irvine, CA, USA) and cloned into T-vectors for Sanger sequencing. The other PCR templates of *E. coli* rRNA and other genes were also prepared by PCR by Q5 DNA polymerase followed by gel purification.

### Assembly PCR by synthetic Dpo4-5m

To assemble the 198 bp *tC19Z* gene using two long primers (*tC19Z*-F115 and *tC19Z*-R113) by assembly PCR, two 113 and 115 nt primers with a 30 bp sequence overlap were designed (Supplementary Table S1). No other template was added to the reaction. No enzyme was added in the negative control experiment. After 20 cycles of reaction, products of both the amplified and control groups were treated by exonuclease I (10 U for 5 μl PCR product) at 37 °C for 10 min. To assemble the 198 bp *tC19Z* gene using six short primers, three steps of assembly PCR with primers *tC19Z*-F1 and *tC19Z*-R1 (step 1), primers *tC19Z*-F2 and *tC19Z*-R2 (step 2) and primers *tC19Z*-F3 and *tC19Z*-R3 (step 3) were performed. PCR products from the last step took up ~5% of the volume of the reaction system in the next. The PCR cycle numbers for steps 1, 2 and 3 were 5, 10 and 20, respectively. The PCR products were analyzed by 3% sieving agarose gel electrophoresis and stained by GoldView (Solarbio). The full-length PCR products were purified by the DNA Clean and Concentrator Kit (Zymo Research) and cloned into T-vectors for Sanger sequencing.
