# Supplementary material for: Total chemical synthesis of a thermostable enzyme capable of polymerase chain reaction
Source: Cell Discov. 2017 Feb 28;3:17008–. doi: 10.1038/celldisc.2017.8 (PMC5335361; doi:10.1038/celldisc.2017.8)
Supplement: Supplementary Information [file celldisc20178-s1.pdf]

Supplementary Information

Total chemical synthesis of a thermostable enzyme capable of polymerase chain reaction

Weiliang Xu[1,2,3], Wenjun Jiang[1,3], Jiaxing Wang[2,3], Linping Yu[1,3], Ji Chen[1], Xianyu Liu[1], Lei Liu[2*], Ting F. Zhu[1*]

[1]School of Life Sciences, Tsinghua-Peking Joint Center for Life Sciences, Center for Synthetic and Systems Biology, Ministry of Education Key Laboratory of Bioinformatics, Tsinghua University, Beijing 100084, China

[2]Tsinghua-Peking Joint Center for Life Sciences, Ministry of Education Key Laboratory of Bioorganic Phosphorus Chemistry and Chemical Biology, Department of Chemistry, Tsinghua University, Beijing 100084, China

[3]These authors contributed equally to this work.

[*]To whom correspondence should be addressed. E-mail: tzhu@biomed.tsinghua.edu.cn (T.F.Z.); lliu@mail.tsinghua.edu.cn (L.L.)

Table of Contents Page

Materials 2
Supplementary Methods 3
Supplementary Figures S1-S16 4
Supplementary Tables S1-S2 20
Supplementary References 22

Materials

Fmoc-amino acids were purchased from GL Biochem (Shanghai, China) and C S Bio (Shanghai, China). Boc-Cys(Trt)-OH, Boc-Cys(Acm)-OH, *O*-(6-chlorobenzotriazol-1-yl)-*N*,*N*,*N'*,*N'*-tetramethyluronium hexafluorophosphate (HCTU) and 1-hydroxybenzotriazole (HOBt) anhydrous were purchased from GL Biochem (Shanghai, China). *N*,*N'*-Diisopropylcarbodiimide (DIC), ethyl cyanoglyoxylate-2-oxime (Oxyma) and DL-1,4-dithiothreitol (DTT) were purchased from Adamas Reagent (Shanghai, China). *N*,*N*-Dimethylformamide (DMF), trifluoroacetic acid (TFA), triisopropylsilane (TIPS), 1,2-ethanedithiol (EDT), thioanisole, sodium 2-mercaptoethanesulfonate (MESNa), $PdCl_2$ and 2,2'-azobis[2-(2-imidazolin-2-yl)propane] dihydrochloride (VA-044) were purchased from J&K Scientific (Beijing, China). *N*,*N*-Diisopropylethylamine (DIEA) was purchased from Ouhe Technology (Beijing, China). Dichloromethane (DCM) and $NaNO_2$ were purchased from Beijing Chemical Works (Beijing, China). $Na_2HPO_4 \cdot 12H_2O$, piperidine, Et_2O, acetic acid and silver acetate were purchased from Sinopharm Chemical Reagent (Shanghai, China). $NaH_2PO_4 \cdot 2H_2O$, NaOH, guanidine hydrochloride (Gn·HCl), hydrochloric acid and NaCl were purchased from Sinopharm Chemical Reagent (Beijing, China). 4-Mercaptophenylacetic acid (MPAA) was purchased from Alfa Aesar (Heysham, England). Tris(2-carboxyethyl)phosphine hydrochloride (TCEP·HCl) was purchased from Tianjin Liankuan Fine Chemicals (Tianjin, China). Glutathione reduced (GSH) was purchased from Acros Organics (Belgium). CH_3CN (HPLC grade) was purchased from J. T. Baker (New Jersey, U.S.).

Supplementary Methods

RP-HPLC and ESI-MS

RP-HPLC analysis and purification were performed by Shimadzu Prominence HPLC systems (solvent delivery units: LC-20AT). C4 and C18 columns (Welch Materials, Shanghai, China) were used. Solvents used were H_2O and CH_3CN (both with 0.1% TFA). The flow rates were set to1 ml/min and 4-6 ml/min for analysis and purification, respectively. ESI-MS spectra were obtained by a Shimadzu LCMS-2020 liquid chromatograph mass spectrometer.

Peptide sequencing by LC-MS/MS

The synthetic Dpo4-5m was purified by 12% SDS-PAGE. In-gel digestion was performed by adding trypsin or pepsin at 37 ℃ overnight, after which the peptides were extracted and analyzed by LC-MS. The samples were separated by gradient elution at a flow rate of 0.3 μl/min by the Thermo-Dionex Ultimate 3000 HPLC system, directly interfaced with a Thermo Scientific Q Exactive mass spectrometer. The analytical column was a home-made fused silica capillary column (75 μm ID, 150 mm length; Upchurch, Oak Harbor, WA, U.S.) packed with C-18 resin (300 Å, 5 μm, Varian, Lexington, MA, U.S.). The Q Exactive mass spectrometer operated under data-dependent acquisition mode by the Xcalibur 2.1.2 software with a single full-scan mass spectrum in the Orbitrap (300-1800 m/z, 70,000 resolution) followed by 20 data-dependent MS/MS scans at 27% normalized collision energy.

Supplementary Figures

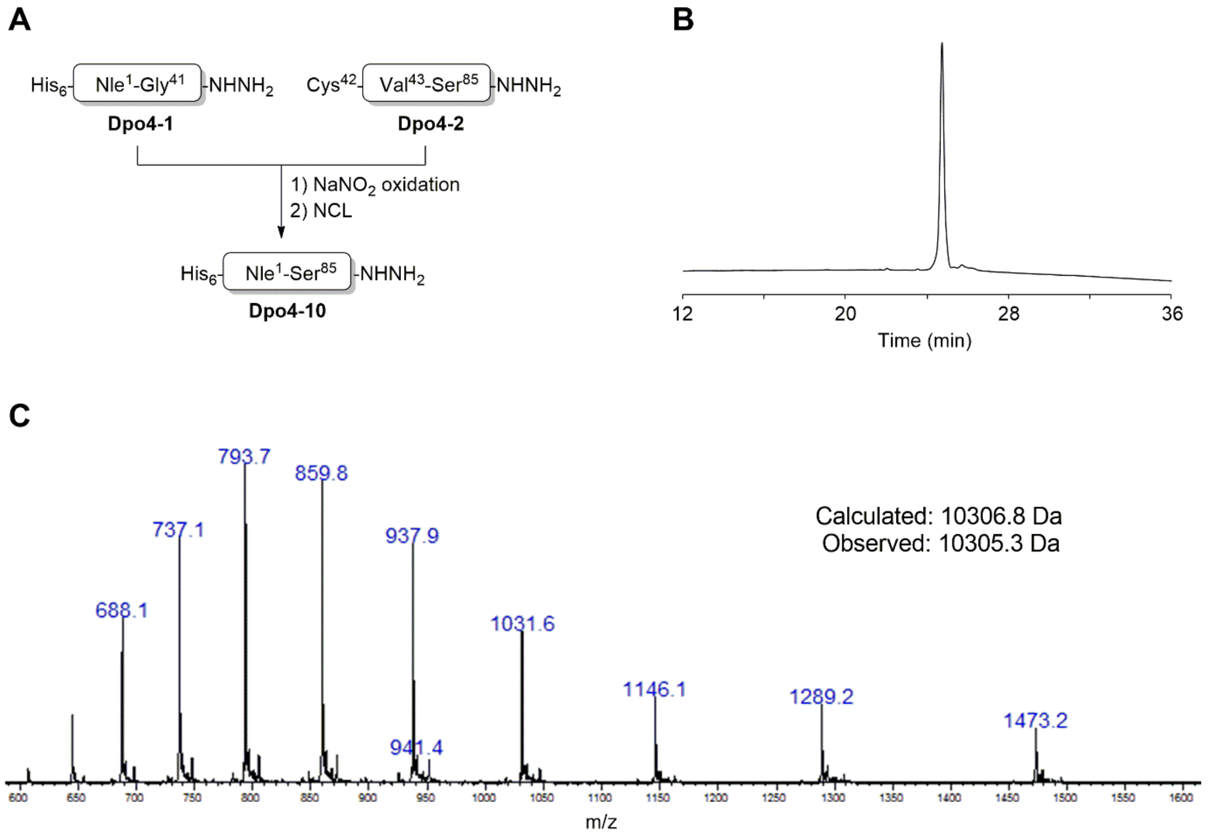

Supplementary Figure S1 | Preparation of Dpo4-10. (**A**) Dpo4-1 (54 mg) was dissolved in 2 ml acidified ligation buffer (aqueous solution of 6 M Gn·HCl and 0.1 M NaH_2PO_4, pH 3.0). The mixture was cooled in ice-salt bath, and 200 μl 0.5 M $NaNO_2$ (in acidified ligation buffer) was added. The reaction was kept in ice-salt bath under stirring for 30 min, after which 2 ml 0.2 M MPAA (in 6 M Gn·HCl and 0.1 M Na_2HPO_4, pH 6.0) was added. After the addition of Dpo4-2 (49 mg), the pH of the reaction mixture was adjusted to 6.5 with NaOH solution at room temperature. After 15 h, the reaction mixture was reduced by TCEP and purified by HPLC (purification conditions: 20-80% CH_3CN gradient (with 0.1% TFA) in H_2O (with 0.1% TFA) over 30 min on a Welch C4 column). Dpo4-10 was obtained with a yield of 51% (52 mg). (**B**) Analytical HPLC chromatogram of Dpo4-10 (λ=214 nm). Column: Welch C4. Gradient: 20-70% CH_3CN (with 0.1% TFA) in H_2O (with 0.1% TFA) over 30 min. (**C**) ESI-MS spectrum of Dpo4-10.

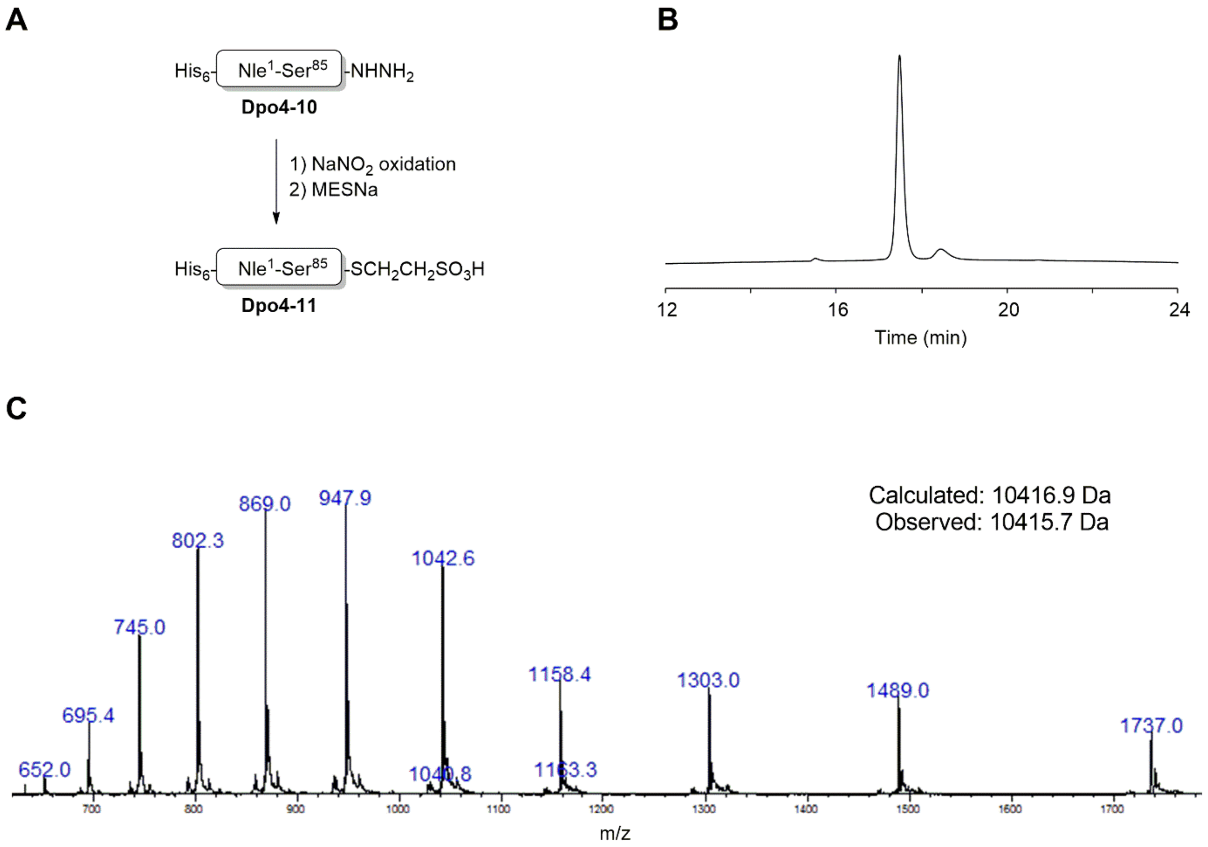

Supplementary Figure S2 | Preparation of Dpo4-11. (**A**) Dpo4-10 (43 mg) was dissolved in 2 ml acidified ligation buffer (aqueous solution of 6 M Gn·HCl and 0.1 M NaH_2PO_4, pH 3.0). The mixture was cooled in ice-salt bath, and 160 μl 0.5 M $NaNO_2$ (in acidified ligation buffer) was added. The reaction was kept in ice-salt bath under stirring for 25 min, after which 24 mg MESNa was added. The pH of the reaction mixture was adjusted to 5.1 with NaOH solution at room temperature. After 1 h, the products were purified by HPLC (purification conditions: 20-70% CH_3CN gradient (with 0.1% TFA) in H_2O (with 0.1% TFA) over 30 min on a Welch C4 column). Dpo4-11 was obtained with a yield of 60% (25 mg). (**B**) Analytical HPLC chromatogram of Dpo4-11 (λ=214 nm). Column: Welch C4. Gradient: 20-100% CH_3CN (with 0.1% TFA) in H_2O (with 0.1% TFA) over 30 min. (**C**) ESI-MS spectrum of Dpo4-11.

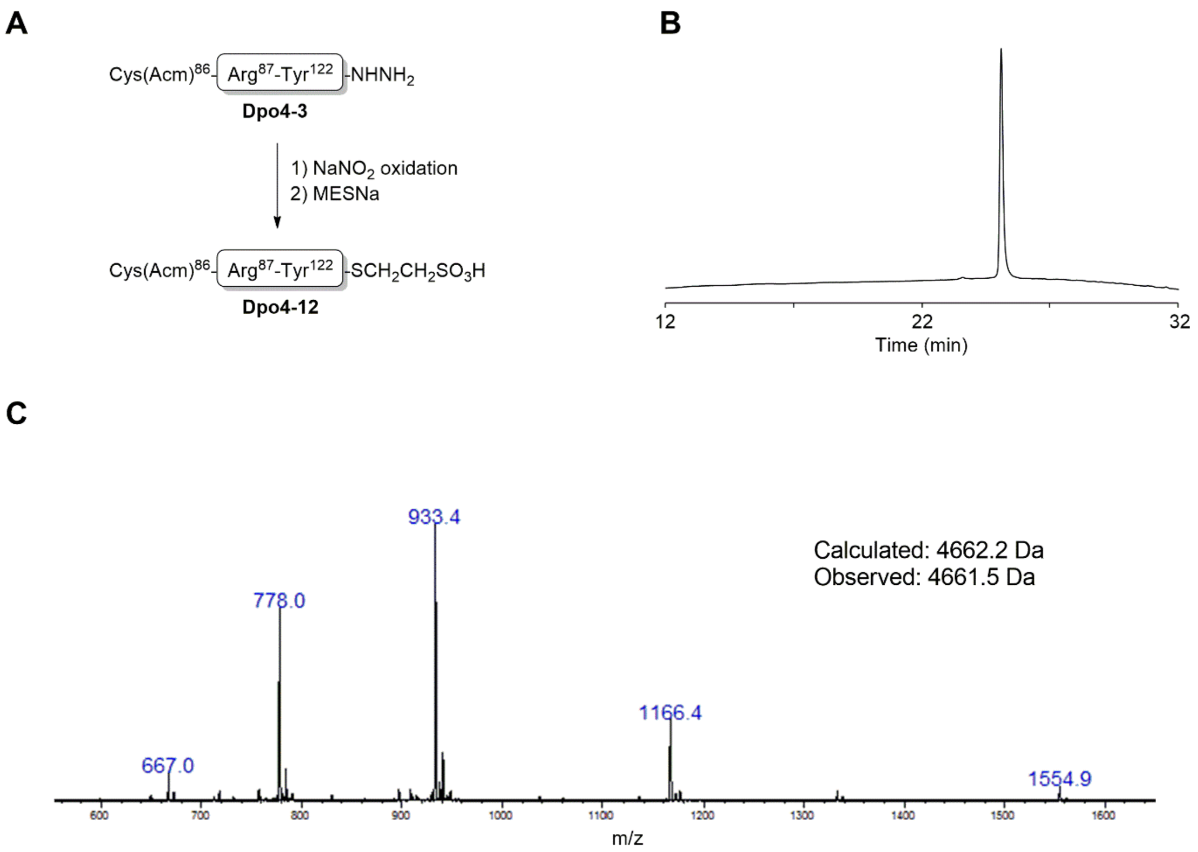

Supplementary Figure S3 | Preparation of Dpo4-12. (**A**) Dpo4-3 (45 mg) was dissolved in 4 ml acidified ligation buffer (aqueous solution of 6 M Gn·HCl and 0.1 M NaH_2PO_4, pH 3.0). The mixture was cooled in ice-salt bath, and 200 μl 0.5 M $NaNO_2$ (in acidified ligation buffer) was added. The reaction was kept in ice-salt bath under stirring for 25 min, after which 1 ml 0.4 M MESNa (in acidified ligation buffer) was added. The pH of the reaction mixture was adjusted to 5.1 with NaOH solution at room temperature. After 1 h, the products were purified by HPLC (purification conditions: 20-70% CH_3CN gradient (with 0.1% TFA) in H_2O (with 0.1% TFA) over 30 min on a Welch C4 column). Dpo4-12 was obtained with a yield of 41% (19 mg). (**B**) Analytical HPLC chromatogram of Dpo4-12 (λ=214 nm). Column: Welch C4. Gradient: 20-70% CH_3CN (with 0.1% TFA) in H_2O (with 0.1% TFA) over 30 min. (**C**) ESI-MS spectrum of Dpo4-12.

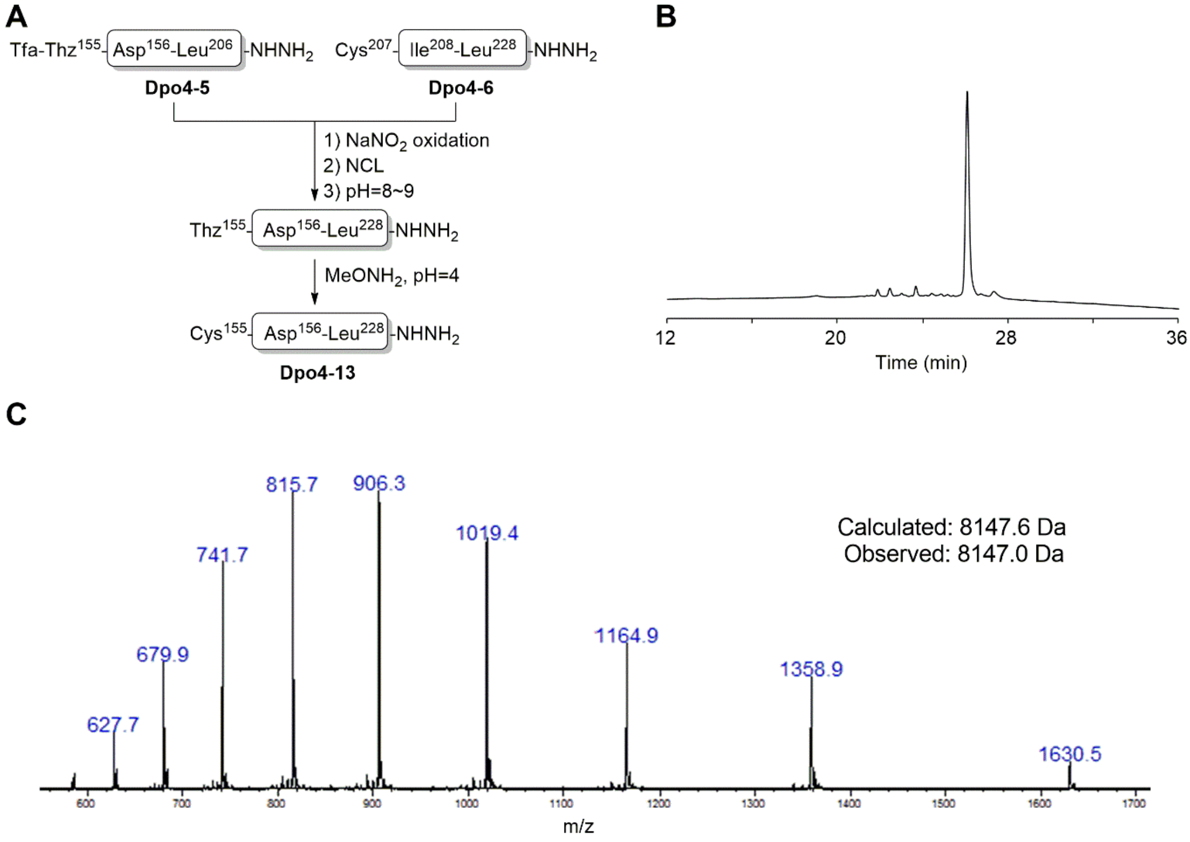

Supplementary Figure S4 | Preparation of Dpo4-13. (**A**) Dpo4-5 (70 mg) was dissolved in 2.4 ml acidified ligation buffer (aqueous solution of 6 M Gn·HCl and 0.1 M NaH_2PO_4, pH 2.9). The mixture was cooled in ice-salt bath, and 240 μl 0.5 M $NaNO_2$ (in acidified ligation buffer) was added. The reaction was kept in ice-salt bath under stirring for ~30 min, after which 2.4 ml 0.2 M MPAA (in 6 M Gn·HCl and 0.1 M Na_2HPO_4, pH 4.9) was added. The pH of the reaction mixture was adjusted to 5.4 with NaOH solution at room temperature. After the addition of Dpo4-6 (36 mg), the pH of the reaction mixture was further adjusted to 6.6. After 11 h, the products were analyzed and the pH was adjusted to 9.0 to promote the complete removal of the Tfa group. After ~1 h, the products were analyzed, and $MeONH_2$·HCl (72 mg) was added to carry out the conversion of Thz into Cys. TCEP·HCl was added until the pH of the reaction mixture reached 4.0. After 3 h, the products were analyzed and purified by HPLC (purification conditions: 20-80% CH_3CN gradient (with 0.1% TFA) in H_2O (with 0.1% TFA) over 30 min on a Welch C4 column). Dpo4-13 was obtained with a yield of 57% (56 mg). (**B**) Analytical HPLC chromatogram of Dpo4-13 (λ=214 nm). Column: Welch C4. Gradient: 20-70% CH_3CN (with 0.1% TFA) in H_2O (with 0.1% TFA) over 30 min. (**C**) ESI-MS spectrum of Dpo4-13.

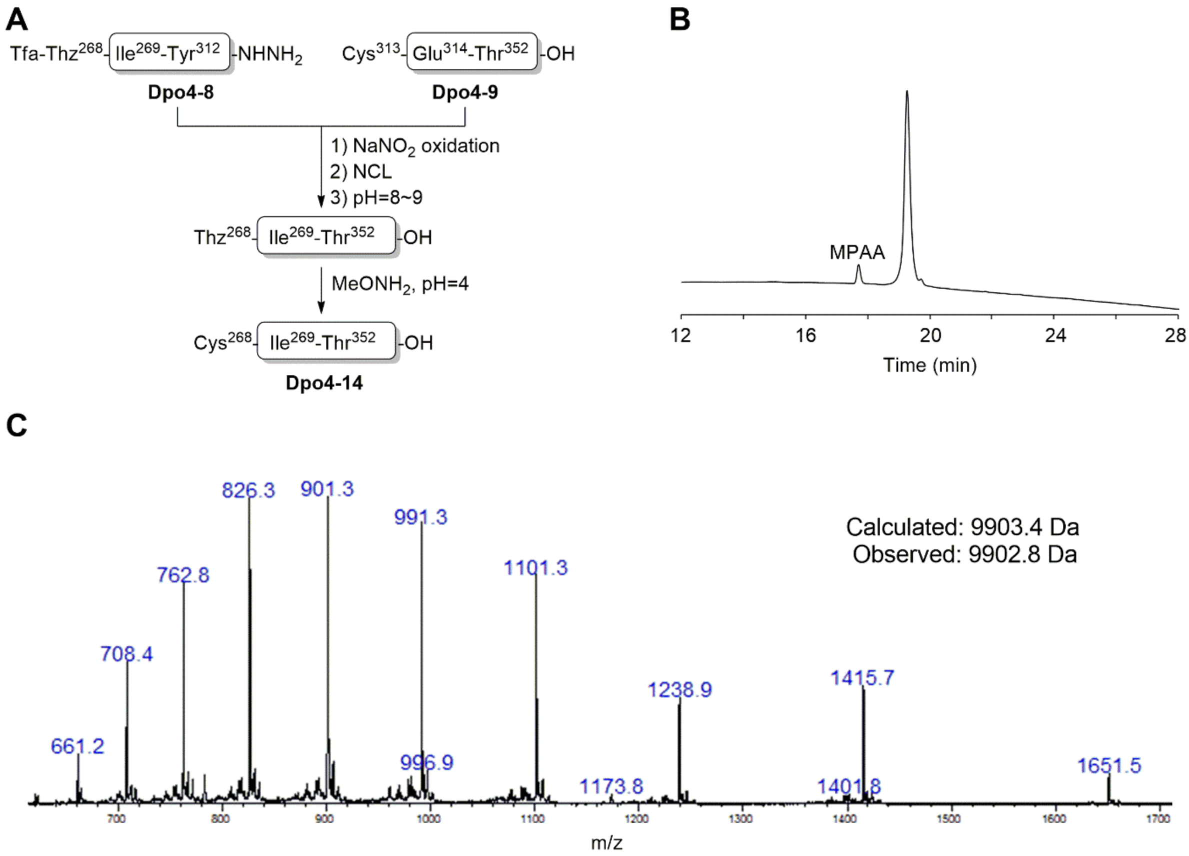

Supplementary Figure S5 | Preparation of Dpo4-14. (**A**) The ligation of Dpo4-8 (53 mg) with Dpo4-9 (48 mg) was carried out following a procedure similar to the ligation of Dpo4-5 with Dpo4-6 (see Supplementary Figure S4). Purification conditions: 10-100% CH_3CN gradient (with 0.1% TFA) in H_2O (with 0.1% TFA) over 30 min on a Welch C4 column. Dpo4-14 was obtained with a yield of 46% (46 mg). (**B**) Analytical HPLC chromatogram of Dpo4-14 (λ=214 nm). Column: Welch C4. Gradient: 20-100% CH_3CN (with 0.1% TFA) in H_2O (with 0.1% TFA) over 30 min. (**C**) ESI-MS spectrum of Dpo4-14.

A

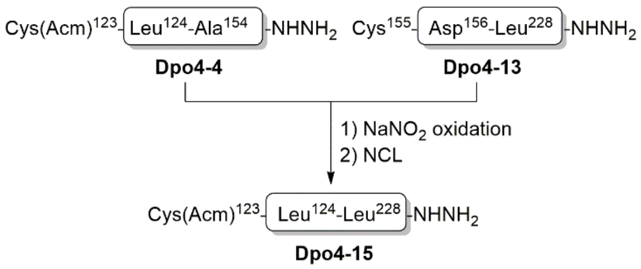

B

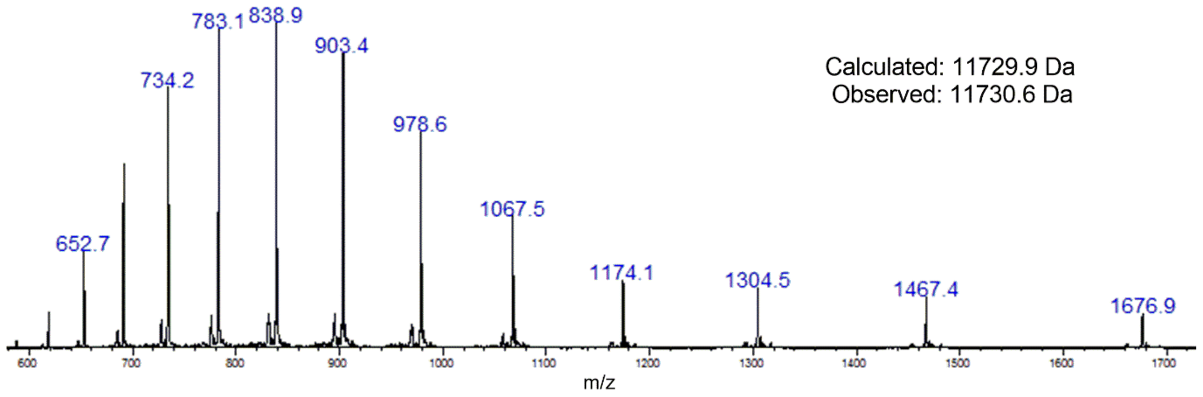

Supplementary Figure S6 | Preparation of Dpo4-15. (**A**) Dpo4-4 (36 mg) was dissolved in 2 ml acidified ligation buffer (aqueous solution of 6 M Gn·HCl and 0.1 M NaH_2PO_4, pH 3.0). The mixture was cooled in ice-salt bath, and 400 µl 0.5 M $NaNO_2$ (in acidified ligation buffer) was added. The reaction was kept in ice-salt bath under stirring for 30 min, after which 2 ml 0.2 M MPAA (in 6 M Gn·HCl and 0.1 M Na_2HPO_4, pH 6.0) was added. After the addition of Dpo4-13 (81 mg), the pH of the reaction mixture was adjusted to 6.5 with NaOH solution at room temperature. After 15 h, the reaction mixture was reduced by TCEP and purified by HPLC (purification conditions: 20-70% CH_3CN gradient (with 0.1% TFA) in H_2O (with 0.1% TFA) over 30 min on a Welch C4 column). Dpo4-15 was obtained with a yield of 46% (54 mg). (**B**) ESI-MS spectrum of Dpo4-15.

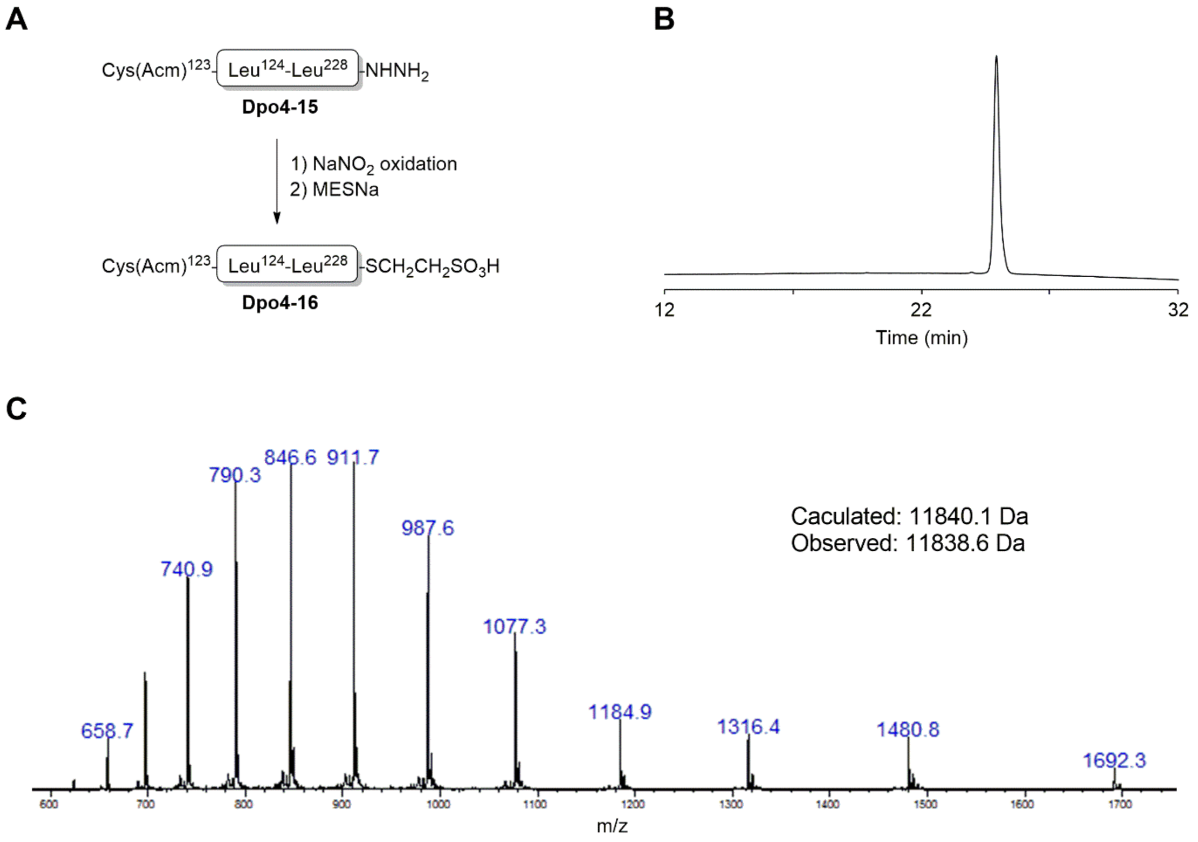

Supplementary Figure S7 | Preparation of Dpo4-16. (**A**) Dpo4-15 (82 mg) was dissolved in 7 ml acidified ligation buffer (aqueous solution of 6 M Gn·HCl and 0.1 M NaH_2PO_4, pH 3.1). The mixture was cooled in ice-salt bath, and 140 μl 0.5 M $NaNO_2$ (in acidified ligation buffer) was added. The reaction was kept in ice-salt bath under stirring for 30 min, after which 1.75 ml 0.8 M MESNa (in acidified ligation buffer) was added. The pH of the reaction mixture was adjusted to 5.5 with NaOH solution at room temperature. After 2 h, the products were analyzed by HPLC and reduced by TCEP before purification (purification conditions: 20-80% CH_3CN gradient (with 0.1% TFA) in H_2O (with 0.1% TFA) over 30 min on a Welch C4 column). Dpo4-16 was obtained with a yield of 83% (69 mg). (**B**) Analytical HPLC chromatogram of Dpo4-16 (λ=214 nm). Column: Welch C4. Gradient: 20-70% CH_3CN (with 0.1% TFA) in H_2O (with 0.1% TFA) over 30 min. (**C**) ESI-MS spectrum of Dpo4-16.

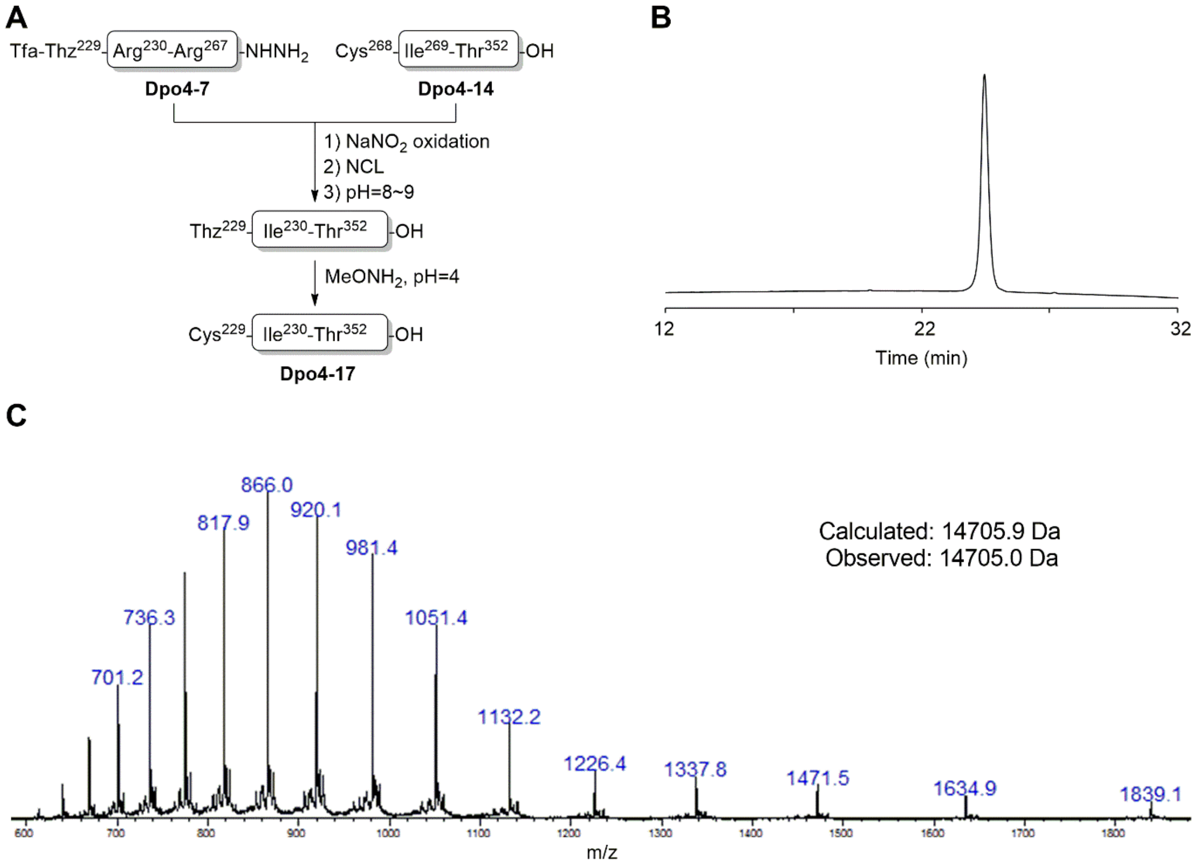

Supplementary Figure S8 | Preparation of Dpo4-17. (**A**) The ligation of Dpo4-7 (25 mg) with Dpo4-14 (47 mg) was carried out following a procedure similar to the ligation of Dpo4-5 with Dpo4-6 (see Supplementary Figure S4). Purification conditions: 20-70% CH_3CN gradient (with 0.1% TFA) in H_2O (with 0.1% TFA) over 30 min on a Welch C4 column. Dpo4-17 was obtained with a yield of 59% (41 mg). (**B**) Analytical HPLC chromatogram of Dpo4-17 (λ=214 nm). Column: Welch C4. Gradient: 20-70% CH_3CN (with 0.1% TFA) in H_2O (with 0.1% TFA) over 30 min. (**C**) ESI-MS spectrum of Dpo4-17.

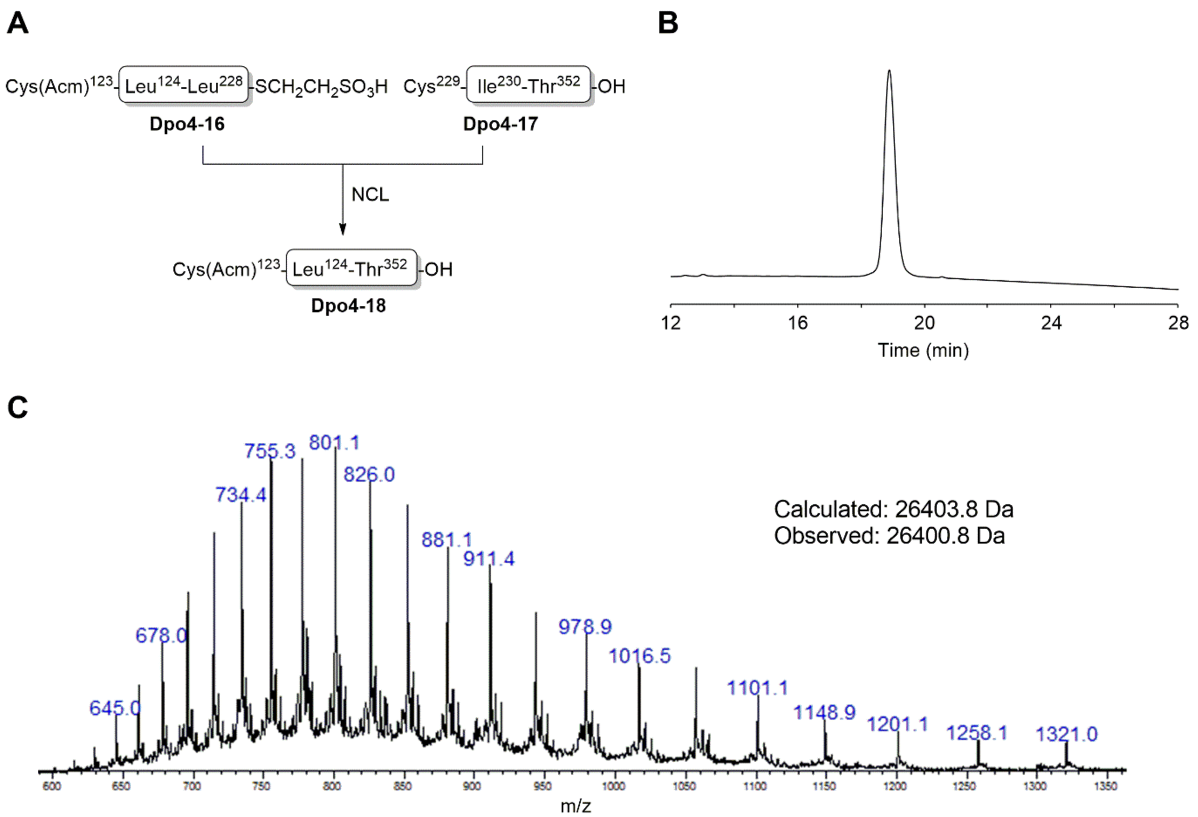

Supplementary Figure S9 | Preparation of Dpo4-18. (**A**) Dpo4-16 (37 mg) and Dpo4-17 (41 mg) were dissolved in a 1.1 ml aqueous solution of 6 M Gn·HCl, 0.1 M Na_2HPO_4, 40 mM TCEP and 125 mM MPAA, pH 6.8. The pH of the reaction mixture was adjusted to 6.6. After 12 h, the products were analyzed by HPLC, after which the products were diluted and purified (purification conditions: 20-80% CH_3CN gradient (with 0.1% TFA) in H_2O (with 0.1% TFA) over 30 min on a Welch C4 column). Dpo4-18 was obtained with a yield of 75% (55 mg). (**B**) Analytical HPLC chromatogram of Dpo4-18 (λ=214 nm). Column: Welch C4. Gradient: 30-80% CH_3CN (with 0.1% TFA) in H_2O (with 0.1% TFA) over 30 min. (**C**) ESI-MS spectrum of Dpo4-18.

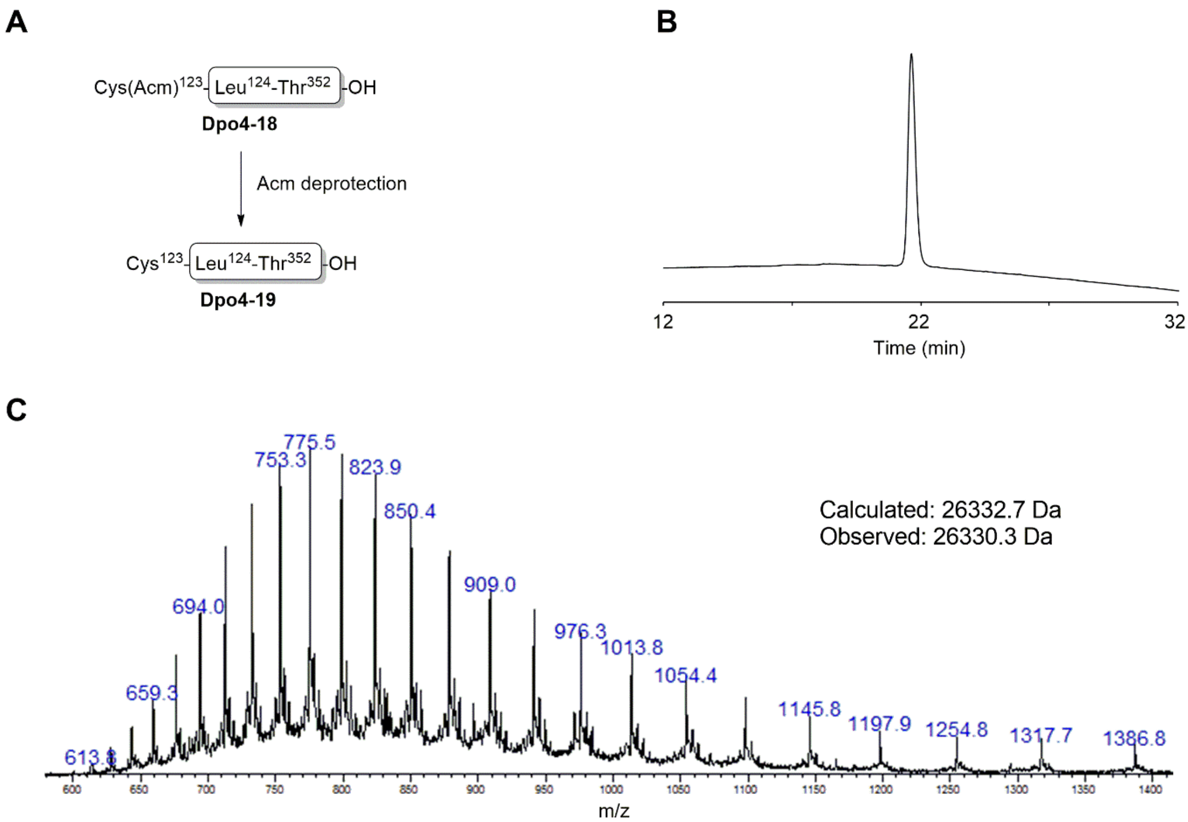

Supplementary Figure S10 | Preparation of Dpo4-19. (**A**) The Acm group in Dpo4-18 was removed by the Pd-assisted deprotection strategy [1]. Dpo4-18 (55 mg) was dissolved in a 2 ml aqueous solution of 6 M Gn·HCl, 0.1 M Na_2HPO_4 and 40 mM TCEP, pH 7.1. $PdCl_2$ (10.4 mg) was dissolved in a 0.4 ml aqueous solution of 6 M Gn·HCl and 0.1 M Na_2HPO_4 and added to the peptide solution. After 13 h, 4 ml 0.75 M DTT (in an aqueous solution of 6 M Gn·HCl and 0.1 M Na_2HPO_4) was added. The reaction mixture was under stirring for 1 h and purified by HPLC (purification conditions: 20-80% CH_3CN gradient (with 0.1% TFA) in H_2O (with 0.1% TFA) over 30 min on a Welch C4 column). Dpo4-19 was obtained with a yield of 86% (47 mg). (**B**) Analytical HPLC chromatogram of Dpo4-19 (λ=214 nm). Column: Welch C4. Gradient: 30-80% CH_3CN (with 0.1% TFA) in H_2O (with 0.1% TFA) over 30 min. (**C**) ESI-MS spectrum of Dpo4-19.

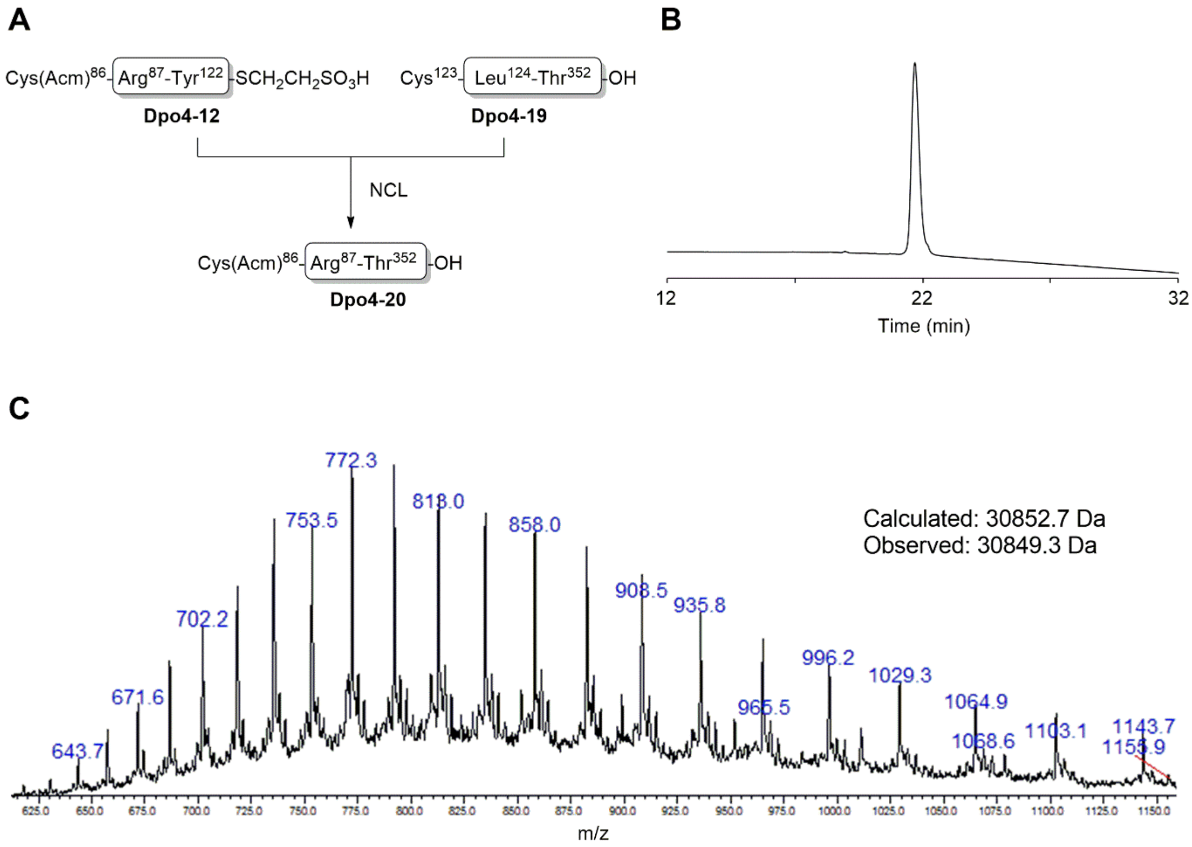

Supplementary Figure S11 | Preparation of Dpo4-20. (**A**) Dpo4-12 (19 mg) and Dpo4-19 (47 mg) were dissolved in a 1.8 ml aqueous solution of 6 M Gn·HCl, 0.1 M Na_2HPO_4, 40 mM TCEP and 100 mM MPAA, pH 6.9. The pH of the reaction mixture was adjusted to 6.6. After 15 h, the products were analyzed by HPLC, diluted, and purified (purification conditions: 20-80% CH_3CN gradient (with 0.1% TFA) in H_2O (with 0.1% TFA) over 30 min on a Welch C4 column). Dpo4-20 was obtained with a yield of 85% (47 mg). (**B**) Analytical HPLC chromatogram of Dpo4-20 (λ=214 nm). Column: Welch C4. Gradient: 30-80% CH_3CN (with 0.1% TFA) in H_2O (with 0.1% TFA) over 30 min. (**C**) ESI-MS spectrum of Dpo4-20.

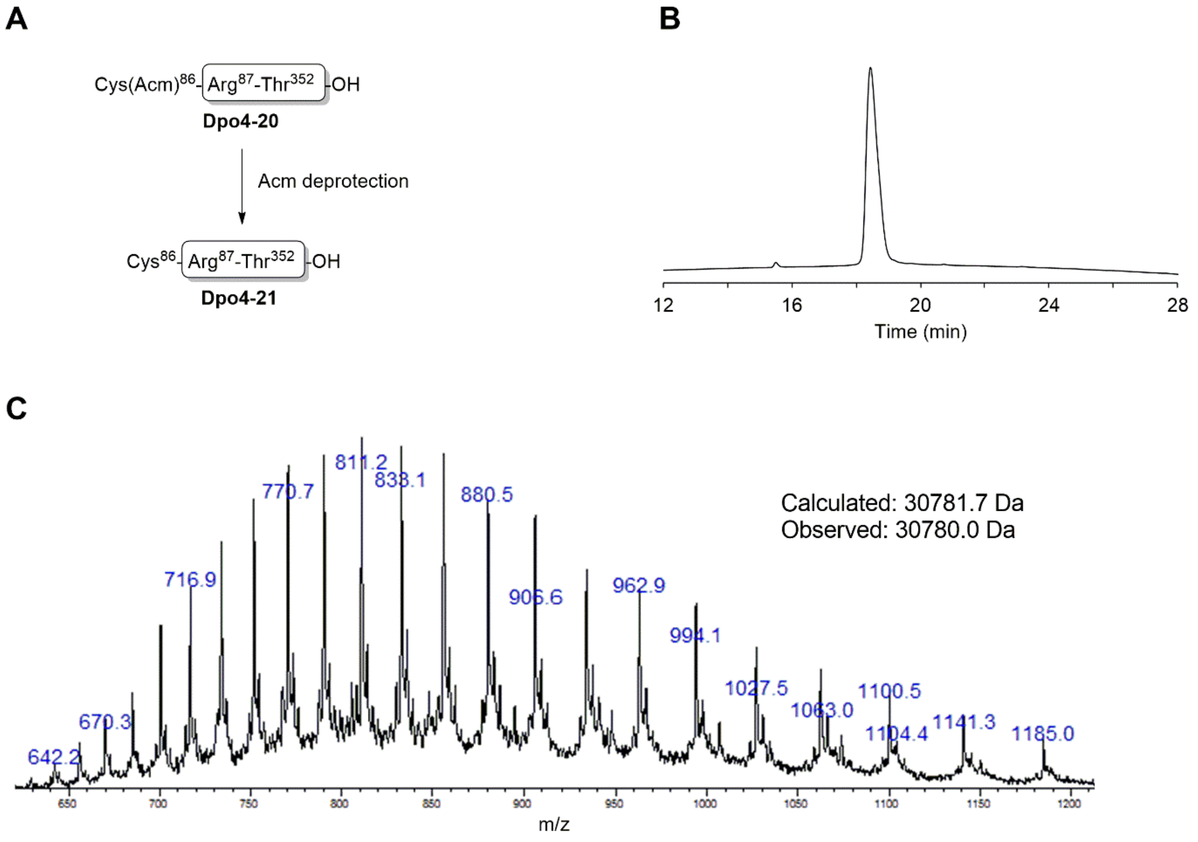

Supplementary Figure S12 | Preparation of Dpo4-21. (**A**) Dpo4-20 (24 mg) was dissolved in a 1.6 ml 50% acetic acid aqueous solution. Subsequently, 10 mg silver acetate was added to the solution. The reaction was under stirring overnight. After 14 h, 0.3 ml 2-Mercaptoethanol was added. The system was diluted to 2-fold with ligation buffer (6 M Gn·HCl, 0.1 M Na_2HPO_4, pH=7). After centrifugation, the supernatant was purified by semi-preparative HPLC. The precipitant was washed thoroughly and purified (purification conditions: 1-70% CH_3CN gradient (with 0.1% TFA) in H_2O (with 0.1% TFA) over 30 min on a Welch C4 column). After lyophilization, the Acm removed product Dpo4-21 was obtained with an 83% yield (20 mg). (**B**) Analytical HPLC chromatogram of Dpo4-21 (λ=214 nm). Column: Welch C4. Gradient: 20-100% CH_3CN (with 0.1% TFA) in H_2O (with 0.1% TFA) over 30 min. (**C**) ESI-MS spectrum of Dpo4-21.

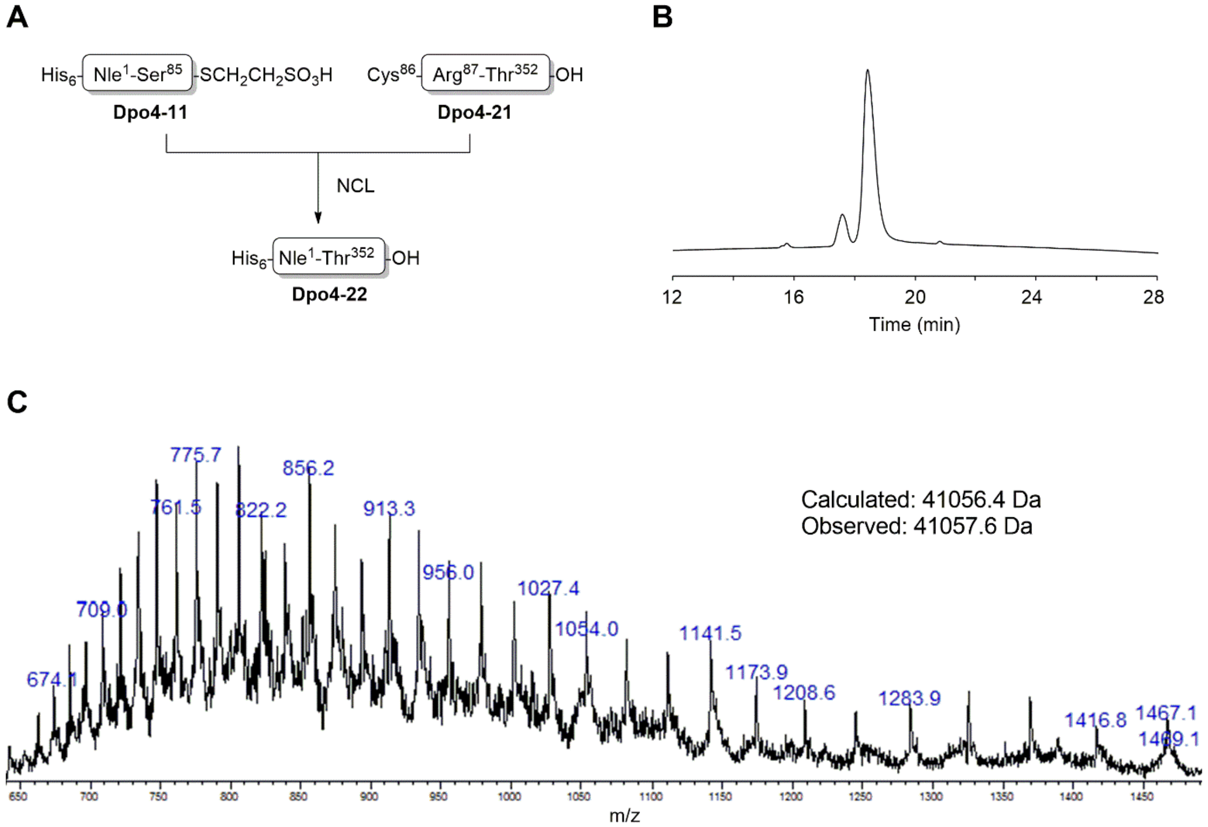

Supplementary Figure S13 | Preparation of Dpo4-22. (**A**) Dpo4-11 (13 mg; 2 eq.) and Dpo4-21 (20 mg) were dissolved in a 0.4 ml aqueous solution of 6 M Gn·HCl, 0.1 M Na_2HPO_4, 40 mM TCEP and 100 mM MPAA, pH 6.9. The pH of the reaction mixture was adjusted to 6.6. After 15 h, the products were analyzed by HPLC, diluted, and purified (purification conditions: 30-80% CH_3CN gradient (with 0.1% TFA) in H_2O (with 0.1% TFA) over 30 min on a Welch C4 column). Dpo4-22 was obtained with a yield of 78% (21 mg). (**B**) Analytical HPLC chromatogram of Dpo4-22 (λ=214 nm). Column: Welch C4. Gradient: 20-100% CH_3CN (with 0.1% TFA) in H_2O (with 0.1% TFA) over 30 min. (**C**) ESI-MS spectrum of Dpo4-22.

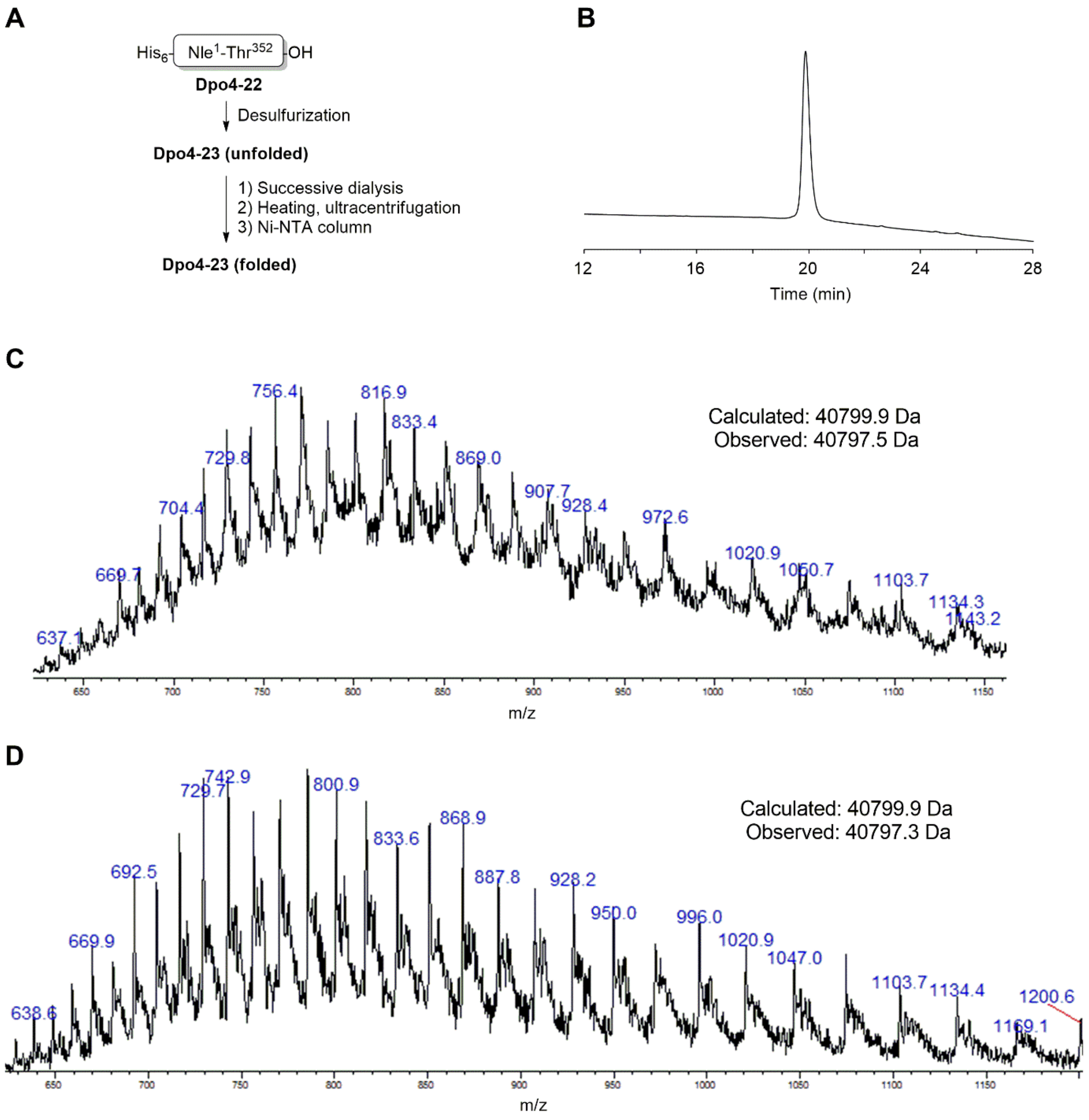

Supplementary Figure S14 | Preparation of Dpo4-23 (Dpo4-5m). (**A**) Dpo4-22 (20 mg) was dissolved in 6 ml 200 mM TCEP solution (6 M Gn·HCl and 0.2 Na_2HPO_4, pH 6.9), with 0.1 mmol (32 mg) VA-044 and 0.2 mmol (62 mg) reduced L-glutathione added. The reaction was under stirring overnight at 37 °C. The desulfurization product Dpo4-23 was analyzed by HPLC and ESI-MS and purified by semi-preparative HPLC (purification conditions: 30-80% CH_3CN gradient (with 0.1% TFA) in H_2O (with 0.1% TFA) over 30 min on a Welch C4 column). After lyophilization, product Dpo4-23 was obtained with a yield of 80% (~16 mg). The desulfurization product then underwent successive dialysis, precipitation of thermolabile peptides, purification by Ni-NTA column, and concentration by centrifugal filter, until the folded synthetic Dpo4-5m with a yield of 15% (~2 mg) was obtained. (**B**) Analytical HPLC chromatogram of the desulfurization product Dpo4-23 (λ=214 nm). Column: Welch C4. Gradient: 20-100% CH_3CN (with 0.1% TFA) in H_2O (with 0.1% TFA) over 30 min. (**C**) ESI-MS spectrum of unfolded synthetic Dpo4-5m. (**D**) ESI-MS spectrum of folded and Ni-NTA column purified synthetic Dpo4-5m.

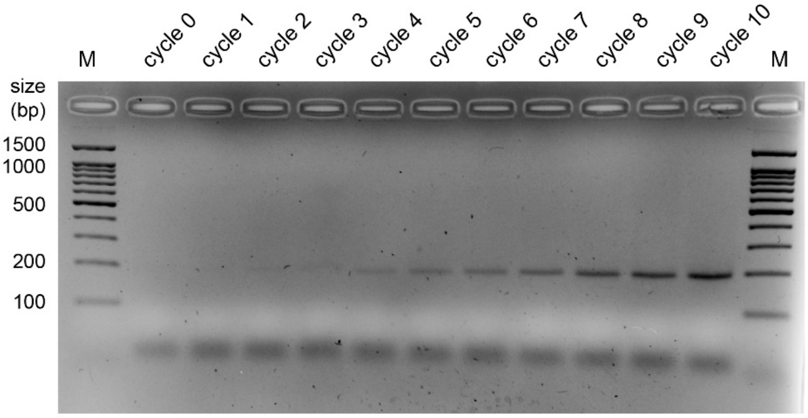

Supplementary Figure S15 | PCR amplification of a 200 bp sequence by synthetic Dpo4-5m, sampled from 0-10 cycles. The products were analyzed by 2% agarose gel electrophoresis and stained by GoldView, with cycle numbers from which they were sampled indicated above the lanes. The PCR amplification efficiency measured ~1.5, which was estimated based on the intensity of the product bands, analyzed by the Image Lab software (Bio-Rad). M, DNA marker.

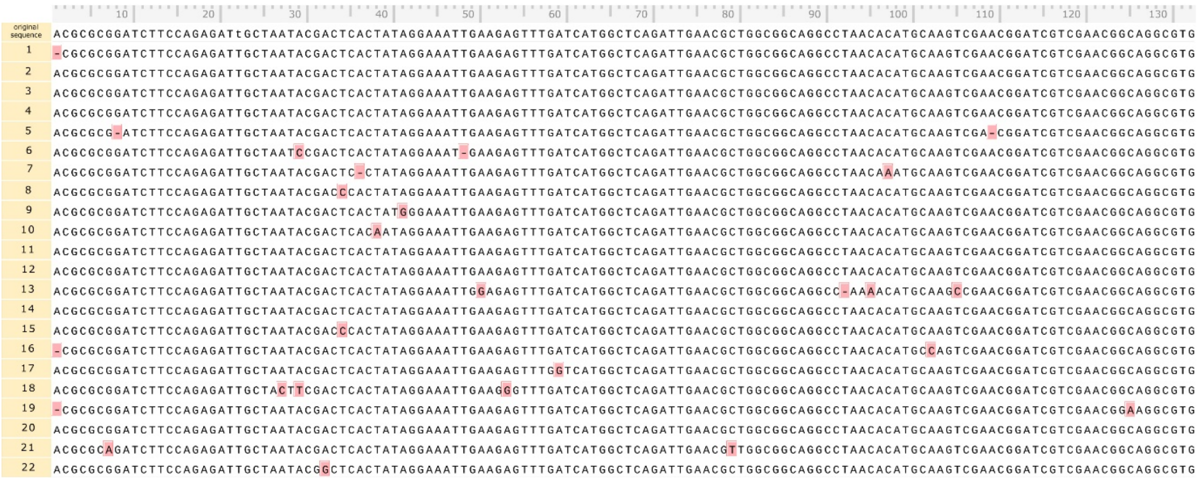

Supplementary Figure S16 | Alignment of Sanger-sequenced PCR amplicons with original template sequence. In total, 22 colonies were cloned into T-vectors for Sanger sequencing. A total of 7 single-base deletions and 19 single-base mutations were present, corresponding to a ~0.9% accumulated mutation rate after 35 cycles.

Supplementary Table S1 | Primer sequences

Primer	Sequence
M13-long-F	5'- GTAAAACGACGGCCAGTGAATTAGAACTCGGT-3'
M13-long-R	5'- CAGGAAACAGCTATGACCATGATTACGCCAAGTTT-3'
5s rRNA(*rrfB*)-F	5'-TGCCTGGCGGCAGTAGCGC-3'
5s rRNA(*rrfB*)-R	5'-ATGCCTGGCAGTTCCCTACTCTCGC-3'
16s rRNA(*rrsC*)-F	5'-AAATTGAAGAGTTTGATCATGGCTCAGATTGAACGCTGG-3'
16s rRNA(*rrsC*)-R	5'-TAAGGAGGTGATCCAACCGCAGGTTCC-3'
dpo4-F	5'-ATGATTGTTCTTTTCGTTGATTTTGACTACTTT-3'
dpo4-R	5'- AGTATCGAAGAACTTGTCTAATCCTATTGCT-3'
tC19Z-F115	5'-GTCATTGAAAAAAAAAGACAAATCTGCCCTCAGAGCTTGAGAACATCTTCGGATGCAGAGGAGGCAGCCTTCGGTGGCGCGATAGCGCCAACGTTCTCAACAGACACCCAATACT-3'
tC19Z-R113	5'-GGAGCCGAAGCTCCGGGGATTATGACCTGGGCGTGTCTAACATCGCCTTTTCGTCAGGTGTTATCCCCACCCGCCGAAGCGGGAGTATTGGGTGTCTGTTGAGAACGTTGGCG-3'
tC19Z-F1	5'-AGAGGAGGCAGCCTTCGGTGGCGCGATAGCGCCAACGTTCTCAACAGACACCCAATACT-3'
tC19Z-R1	5'-CAGGTGTTATCCCCACCCGCCGAAGCGGGAGTATTGGGTGTCTGTTGAGAACGTTGGCG-3'
tC19Z-F2	5'-CAAATCTGCCCTCAGAGCTTGAGAACATCTTCGGATGCAGAGGAGGCAGCCTTCGGTGG-3'
tC19Z-R2	5'-ATTATGACCTGGGCGTGTCTAACATCGCCTTTTCGTCAGGTGTTATCCCCACCCGCCGA-3'
tC19Z-F3	5'-GTCATTGAAAAAAAAAGACAAATCTGCCCTCAGAGCTTGAGAACATCTTCG-3'
tC19Z-R3	5'-GGAGCCGAAGCTCCGGGGATTATGACCTGGGCGTGTCTAACATCGCC-3'

Supplementary Table S2 | Synthetic protein sequencing

The aa sequence of the synthetic protein was validated by liquid chromatography–tandem mass spectrometry (LC-MS/MS), with a collective 100% coverage from two independent experiments with the peptide treated by trypsin and pepsin, respectively. See the Excel file for results.

Supplementary References

1. Maity SK, Jbara M, Laps S, Brik A. Efficient Palladium-Assisted One-Pot Deprotection of (Acetamidomethyl)Cysteine Following Native Chemical Ligation and/or Desulfurization To Expedite Chemical Protein Synthesis. *Angewandte Chemie* 2016; **55**(28): p. 8108-12.
